# The Achilles Heel of Protein Biochemistry: Insolubility of Recombinant Proteins—A Case Study About Producing a Rice Enzyme

**DOI:** 10.3390/ijms26188974

**Published:** 2025-09-15

**Authors:** Tibo De Coninck, Hannes Vanhaeren, Els J. M. Van Damme

**Affiliations:** Laboratory for Biochemistry and Glycobiology, Department of Biotechnology, Ghent University, Proeftuinstraat 86, 9000 Ghent, Belgium; tibo.deconinck@hotmail.com (T.D.C.); hannes.vanhaeren@ugent.be (H.V.)

**Keywords:** recombinant proteins, protein solubility, *E. coli*, *P. pastoris*, *A. thaliana*, OsAPSE

## Abstract

Biochemical characterization of proteins is fundamental to understanding their function. Typically, research in protein structure/function requires reasonable quantities of the protein of interest. Because of the low abundances in their natural environment, the heterogenous state of post-translational modifications, and the difficulty of obtaining the tissue containing the protein of interest, recombinant protein production is usually employed. One of the major difficulties impeding advances in biotechnological research is protein insolubility, undermining further downstream research and applications. *Escherichia coli* strains are popular hosts for protein production but are often unfit for the expression of eukaryotic sequences due to the absence of proper post-translational modifications, some of which are crucial for protein folding and activity. Here, we showcase the challenges researchers may be confronted with when trying to produce proteins recombinantly, by using OsAPSE, an enzyme from rice, as an example of a difficult-to-produce protein. Several production hosts were explored, and best results were obtained when OsAPSE was produced in *E. coli* combined with a solubility tag or when a higher eukaryotic system was used. This study highlights common pitfalls in protein research and provides strategies to overcome them, making it a case study for researchers facing similar challenges.

## 1. Introduction

Protein production is generally considered ‘successful’ if reasonable quantities of soluble and active protein of interest (POI) are obtained in a cost-effective and timely manner. Therefore, recombinant protein production is often opted for when expression levels in natural sources are low or when higher protein abundances are required. Protein production in living cells benefits from multiple advantages related to production costs, scalability, yield and downstream processing, depending on the considered system [1] (Supplementary File S1). There are, however, several important constraints to cell-based production strategies, limiting or even impeding successful protein production [2,3,4,5]. In general, production of prokaryotic proteins is confronted with fewer difficulties or challenges compared to production of eukaryotic proteins. Prokaryotic proteins are usually devoid of extensive post-translational modifications (PTMs). The opposite is true for eukaryotic proteins, as these often require *N*/*O*-glycosylation and/or disulfide bridges for oxidative protein folding, activity and solubility [6]. Interestingly, the largest share of recombinant proteins, in industry and in research, are produced using a prokaryotic system [7,8], regardless of the protein’s origin. In fact, the large majority (>80%) of all proteins present in the Protein Database (PDB) have been produced recombinantly in *E. coli* [9], while prokaryotic sequences only make up 36% of the sequences present in PDB.

The intrinsic ability of a protein to dissolve depends on the specific distribution of hydrophilic and hydrophobic residues, as well as the charge distribution, on the protein surface [10]. Aggregation occurs when hydrophobic groups are exposed and mutually interact. Proteins have the intrinsic ability to fold into their native structure, by navigating through a funnel-shaped folding energy landscape. During the folding process, proteins follow different folding pathways, assume various intermediate conformations, which are either energetically (un)favorable along the way to their desired native structure with lowest energy state and fully developed tertiary (and quaternary) structures [11]. Proteins can also assume partially folded states such as the ‘*molten globule state*’, in which secondary structures are largely formed but with less defined tertiary structures [12] or other stable non-native conformations [11]. In these cases, chaperones may help proteins to assume native folding and prevent aggregation by overcoming kinetic and thermodynamic barriers [13]. The folding route of a POI may be negatively inclined towards the misfolded state due to the absence of adequate PTMs, which directly impact protein structure. Misfolded proteins are kinetically and thermodynamically ‘trapped’ within the energy landscape and may aggregate and form so-called ‘inclusion bodies’ (IBs). IBs are typically observed in prokaryotic production systems and comprise insoluble cytosolic aggregated and misfolded proteins, often due to the absence of PTMs or too high biosynthesis rates [14].

Protein insolubility and IB formation are considered one of the most important challenges in protein research. In most cases, IBs are undesired since soluble POI are required for biochemical research or industrial applications [8,14]. Obtaining the POI in IBs may present researchers with unique opportunities as they contain insoluble, but very pure forms of the POI and are easy to isolate through centrifugation. Furthermore, proteins in IBs are not necessarily inactive, but may even contain large quantities of active POI [15]. Unfolding and refolding strategies are widely performed [16], although there are no universal protein refolding protocols. However, obtaining refolded POI is no guarantee for protein activity, as its native structure is not always recovered [17]. Therefore, it is evident why prokaryotic proteins are produced most optimally in prokaryotic hosts and vice versa for eukaryotic proteins and hosts. Hence, it is strongly recommended to make use of the appropriate production system, taking the basic characteristics of the host as well as the inherent characteristics of the POI into account. In contrast to several other protein characteristics, protein solubility is practically impossible to predict [18,19]. Hence, empirical testing remains essential.

One should also consider PTM differences between eukaryotes. For instance, the glycosylation properties of animal proteins are very different from the glycosylation present in yeast, insect or plant cells [20,21]. Although a general rule is to produce prokaryotic proteins in prokaryotes and eukaryotic proteins in eukaryotes, there are many exceptions, thereby emphasizing the unpredictability and uniqueness of every recombinant protein production. In some cases, the absence of endogenous PTMs is desired to remove background noise, necessary to study the processes associated with PTMs. In these cases, it is useful to express eukaryotic sequences in prokaryotic hosts. For instance, phosphorylation of the plant protein kinase BRASSINOSTEROID-INSENSITIVE1 was studied in *E. coli* to provide a PTM-free background, ensuring that any observed phosphorylation originated solely from the kinase itself rather than from host modifications [22].

Because of their ease of transformation, bacteria are usually the first platform of choice, even when the POI is of eukaryotic origin. A plethora of examples demonstrate the successful recombinant production of eukaryotic proteins in prokaryotic hosts [23,24]. When bacterial systems fail to produce a eukaryotic POI, yeasts are usually the next in line to experiment with, although yeasts have important drawbacks, including the phenomenon of hyper-glycosylation. There are, however, yeast strains available that are devoid of this characteristic, or even provide plant-like and/or human-like *N*-glycosylation [25,26]. Other important eukaryotic protein platforms include mammalian cells, insect cells and plant cells (Supplementary File S1).

The protein of interest for this study is OsAPSE, a bifunctional enzyme from Japanese rice (*Oryza sativa* subsp. Japonica). This particular POI was part of an overarching research project aiming for the biochemical characterization and elucidation of the biological function and properties of multi-domain proteins comprising carbohydrate-binding and carbohydrate-cleaving domains from rice and *Arabidopsis thaliana* [27]. Studying the enzymatic and biochemical properties of an enzyme requires reasonable quantities of purified POI, and this particular aspect was especially challenging for OsAPSE. Recently, we reported the biological function of OsAPSE in the developmental process of rice seed germination and were able to demonstrate the enzymatic characteristics of OsAPSE by using a cell-free production system [28]. The cell-free system was used after all traditional cell-based approaches were not successful. This research article specifically focusses on the cell-based strategies that were followed in many attempts to obtain OsAPSE.

Finding the most optimal experimental conditions for recombinant protein production is indeed empirical and known to be a trial-and-error process [8], especially because the multitude of experimental parameters to be varied are endless, rendering the design space virtually infinite. Recombinant protein production is not straightforward and sometimes certain designs or choices are made, prompted by sudden unexpected breakthroughs or leads found in the literature. An important consequence of this approach is that the chosen research path may not appear straightforward from the outset and can take unexpected turns that were not obvious to foresee or to explain in hindsight. Therefore, the attempts to produce a POI recombinantly might look unstructured or chaotic. This article aimed to compile the delivered efforts to produce OsAPSE and its subdomains and includes research endeavors from the period 2019–2024. We aimed for an honest illustration of how difficult and unpredictable recombinant protein production can get. It is not the aim of this article to provide a chronological overview of all experiments conducted, as this would create unnecessary redundancy. In contrast, this article gives an overview of the arising problem of protein solubility, addresses some important breakthroughs and formulates some considerations, hoping that it might be inspiring to researchers facing similar challenges.

## 2. Results

### 2.1. Characteristics of OsAPSE

**Figure 1:**
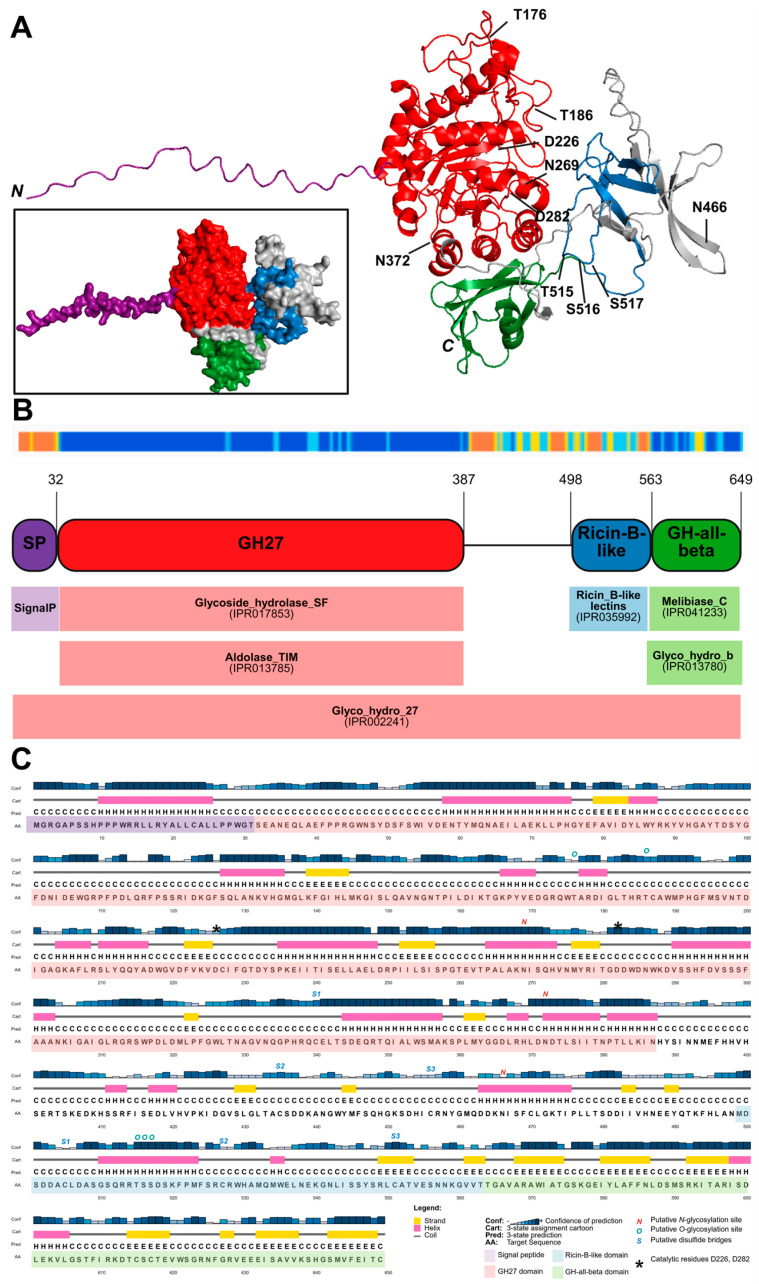
OsAPSE is a multi-domain protein composed of an *N*-terminal signal peptide (purple), a GH27 domain (red), a ricin-B-like domain (blue) and a *C*-terminal GH-all-beta domain (green). The model of OsAPSE was obtained through AlphaFold and was constructed with high confidence (predicted Local Distance Difference Test value = 83.36). (**A**) 3D representation of OsAPSE shown as ribbon diagram and protein surface. The sites for *N*/*O*-glycosylation as well as the catalytic sites are indicated. (**B**) Domain modularity representation of OsAPSE. The confidence of structure prediction is indicated on a sliding scale between orange (low confidence) to blue (high confidence). The domain boundaries and InterPro annotations are shown. (**C**) Primary and secondary structure of OsAPSE, showing strands, helices and coils. The confidence of the secondary structure prediction is shown on a sliding scale between light blue (low confidence) to dark blue (high confidence). Putative *N*/*O*-glycosylation sites as well as cysteines possibly involved in disulfide bridge formation are shown with a red *N*, green *O*, or blue *S*. The cysteine residues from the same disulfide bridges are enumerated with the same number (*S1*, *S2*, *S3*). The catalytic residues D226 and D282 are indicated with asterisks.

OsAPSE is a multi-domain enzyme from Japanese rice (Figure 1A), containing 649 amino acid residues, with a calculated molecular weight of 73.1 kDa and an iso-electrical point at pH = 6.1 [28]. The protein was named after AtAPSE, its homolog in *Arabidopsis thaliana* [29]. The coding sequence of OsAPSE consists of an *N*-terminal signal peptide (residues 1–32), a glycoside hydrolase (GH) family 27 domain (residues 42–387), a ricin-B-like lectin domain (residues 498–563) and a *C*-terminal GH-all-beta domain (residues 563–649) (Figure 1B). In silico predictions (Figure 1C) revealed that the OsAPSE sequence possibly contains 3 sites for *N*-glycosylation, 5 sites for *O*-glycosylation and 3 disulfide bridges. In short, the *N*-terminal signal peptide is of importance for protein secretion under native conditions. The GH27 domain assumes a TIM-barrel fold and confers dual α-D-Galactopyranosidase (AGAL) and β-L-Arabinopyranosidase (ARAP) activity towards substrates with α-D-Gal*p* and β-L-Ara*p* side chains from plant cell wall polysaccharides [28]. The ricin-B-like domain is a key determinant for the phylogeny of OsAPSE in its homologues within the GH27 family. This lectin-like domain is truncated and shows many similarities towards ricin-B lectins as well as proteins with a carbohydrate-binding module of family 13 [30], which typically have a pseudo-symmetric threefold β-trefoil fold [31,32,33,34]. The *C*-terminal GH-all-beta domain is often observed in carbohydrate-active enzymes and confers structural stability and assists in protein folding [35,36].

### 2.2. Expression in E. coli Leads to Mostly Insoluble Proteins

Several experiments making use of diverse expression constructs and experimental conditions have been executed in attempts to obtain soluble OsAPSE (Table 1). Although experiments to optimize the expression conditions (i.e., incubation temperature and duration, concentration of inducer) have also been carried out, these are not reported in this study to limit redundancy. We will focus on the results from experiments 1, 14, 17, 19, 21 and 26 (Table 1). In general, almost every experiment with *E. coli* as a host resulted in the expression of recombinant OsAPSE or its subdomains, being present in the insoluble cytosolic fraction as part of IBs (Supplementary File S2). Supplementary File S2 illustrates the outcomes of OsAPSE expression in different *E. coli* strains. Most constructs yielded insoluble protein, except for the GH27 domain tagged with the MBP tag (experiment 26).

#### 2.2.1. Effect of Codon Optimization

The factors leading to an insoluble protein are not completely understood and considered a ‘black box’, although there is a lot of empirical evidence that certain experimental parameters can improve protein solubility. Very often, proteins are produced recombinantly, using a host organism that differs from the sequence origin. It is widely accepted to produce synthetic DNA sequences for which codon bias between the native organism and the expression host has been taken into account [37]. Codon optimization encompasses substituting native codons according to the codon preference/bias of the production host. Native codon usage is measured by the Relative Synonymous Codon Usage (RSCU) value, which is a percentage, indicating the relative usage of a codon for a particular amino acid. RSCU values are used to calculate the Codon Adaptation Index (CAI) for a particular POI in a particular host. The CAI is normalized against RSCU values of highly expressed proteins, yielding a value between 0–1, where 1 means that the POI is produced with the same ease as highly expressed proteins and 0 means that the POI will not be produced at all [38]. Several reports indicate that taking codon bias into account through codon optimization will significantly enhance protein solubility, which was observed for the production of, for instance, KRAS4B, a human protein associated with MAPK signaling in cancers [37]. The CAI of OsAPSE for expression in *E. coli* equals 0.54, indicating that the non-modified sequence of OsAPSE would be expressed with an efficiency of 54% compared to highly expressed proteins. Analysis of rare codons revealed that the native OsAPSE sequence from rice contained multiple (n = 89; 13.7%) rare codons. After codon optimization, the codon quality improved considerably, reaching a CAI up to 0.89 (Supplementary File S3). The codon-optimized sequence was used throughout all experiments in *E. coli*, except in experiments 17–22 where harmonization, alternative codon optimization algorithms and PROSS were used. However, codon optimization did not improve the solubility of OsAPSE (Supplementary File S2A).

#### 2.2.2. Effect of Codon Harmonization and Mutational Variants

In contrast to codon optimization, codon harmonization encompasses the mimicking of the relative codon frequency of the native gene of interest in the production host, thereby ensuring ‘translational pauses’ that would occur naturally in the native background. Unfortunately, codon harmonization did not yield a soluble POI. Both codon harmonization and optimization yielded insoluble proteins (Table 1), indicating that the solubility issue was more deeply rooted than merely codon usage. Therefore, another strategy, utilizing the PROSS tool to create mutation variants of the codon-optimized OsAPSE sequence was considered. The underlying principle of PROSS is the introduction of multiple wisely considered mutations, based on atomistic modeling and phylogenetic sequence information, possibly resulting in proteins with a more energetically favorable folding state resembling the native state [11,12,39]. The PROSS algorithm has been proven useful in the past for recombinant production of human acetylcholinesterase, histone deacetylase and DNA methyltransferase, and delivered good results with considerably increased expression levels and improved protein stability [40].

We obtained 9 PROSS variants of OsAPSE that differed between 1.3 and 7.2% compared to the original sequence, but were structurally very similar (Table 2). Most common mutations included H92P, N103V, L114P, F117W, G155A, I309P, E341N, S344L/P and S642C. Remarkably, several native residues were substituted by proline residues, which are known to drastically disrupt protein structure by introducing kinks in α-helices [41]. However, the mutations were not applied in regions with secondary structure elements or active sites. The introduction of proline residues did not distort protein structure compared to the native OsAPSE (Table 2). However, the introduction of mutations that could be beneficial for the energy state of the protein folding did not contribute to a solubility shift. It should be noted that PROSS works based on 3D structures of the POI together with the structures of homologs. However, at present, no resolved structures for OsAPSE or its natural homologues exist, thereby reducing the efficacy of PROSS in our case. We used the AlphaFold model of OsAPSE, although this structure contains a highly uncertain region, i.e., the ricin-B-like domain, as indicated in Figure 1.

#### 2.2.3. Utilization of *E. coli* Strains Capable of Synthesizing Disulfide Bridges

The possibility to create disulfide bridges by using the *E. coli* SHuffle^®^ strain did not shift the solubility state of the GH27 domain of OsAPSE (Supplementary File S2B). It was reported before that the use of this strain may lead to variable results in terms of expression level and protein solubility, emphasizing the need for optimizing for each recombinant protein [42].

#### 2.2.4. Usage of Solubility Tags

Experiment 26 was the only one that yielded a soluble recombinant protein for the GH27 domain of OsAPSE, in combination with an *N*-terminal MBP tag and TEV protease cleavage site, and a *C*-terminal 3xFLAG and His_6_ tag (Supplementary File S2D). The combination with the MBP solubility tag resulted in highly abundant protein production in the soluble phase. Interestingly, we noticed an important effect of the cell lysis technique. When lysing *E. coli* cells by using a lysis buffer, the GH27 domain ended up being insoluble, while soluble POI was obtained when using sonication in combination with the TGH1 buffer (Supplementary File S2D). MBP is considered to be one of the most effective fusion tags enhancing protein solubility, although the exact mechanism how MBP increases the solubility of its fusion partner is not completely understood [43,44]. It is hypothesized that MBP possesses chaperone-like properties and stabilizes the folding process of its fusion partner, thereby reducing the likelihood of misfolding and protein aggregation. In addition, MBP by itself is naturally secreted to the periplasm through the general secretory pathway [45,46]. There are also several other solubility enhancing tags available, such as glutathione S-transferase (GST) and thioredoxin (TRX) [47]. Our results indicate that the presence of MBP in itself is not a determinant for OsAPSE solubility, since drastic changes in protein solubility were observed only when the lysis method was changed. We assume that multiple factors are at play that work together and synergistically, including construct design (i.e., position and number of solubility tags), experimental conditions (i.e., production temperature, culture density, inducer concentration) and lysis method [48].

#### 2.2.5. Exploration of Protein Refolding

Because the solubility of OsAPSE remained a bottleneck, protein unfolding and refolding was attempted using a series of refolding buffers. Recombinant OsAPSE was unfolded and obtained a protein concentration of 2.8 mg/mL (Supplementary File S4.1). Best results were obtained for alkaline buffers (i.e., 50 mM bis-Tris pH 9 or 50 mM piperazine pH 10) in combination with 15 mM beta-mercaptoethanol, salts (i.e., 1–20 mM KCl, 20–250 mM NaCl, 5 mM CaCl_2_, 5 mM MgCl_2_), 2 mM glutathione and glutathione disulfide in a 10:1 ratio, 10% (*v*/*v*) glycerol, 400 mM L-arginine, 0.01% (*w*/*v*) PEG-1000 and 0.2% (*v*/*v*) CHAPS (Supplementary File S4.2), although A_405_ was just above the set threshold of 0.05. In experiment 14, unfolding and refolding of the POI was executed by the company GenScript (Supplementary File S2D).

#### 2.2.6. Enzymatic Activity of Soluble GH27_OsAPSE and Refolded OsAPSE

The refolded OsAPSE proteins from experiment 14 and the soluble GH27 domain obtained in experiment 26 were submitted to discontinuous enzymatic assays screening for AGAL activity (Table 1). Activity assays with the non-purified MBP-tagged GH27 domain showed no measurable AGAL activity (Supplementary File S5.1). In fact, the absorbance values decreased over time. In contrast, low AGAL activity was observed when the refolded OsAPSE proteins from experiment 14 were submitted to an enzymatic assay (Supplementary File S5.2). The increase in absorbance over time was dependent on the substrate concentration, as expected for enzymes obeying Michaelis–Menten kinetics. An increase of 53 µOD/s, 84.6 µOD/s, 111.6 µOD/s and 181 µOD/s were observed for the substrate concentrations of 1 mM, 5 mM, 10 mM and 20 mM *p*NP-α-D-Gal*p*, respectively. These observations allowed estimation of the K_M_ and V_max_ value for the protein, based on the linearization methods of Hanes–Woolf and Eadie–Hofstee. Both linearization methods yielded the same maximum velocity and Michaelis constant, V_max_ = 10.3 nM/s and K_M_ = 44 mM, respectively. The reported V_max_ value is mainly depending on the enzyme concentration used (i.e., 50 µg/mL~65.5 nM). The calculated value for K_M_ is considerably higher than that observed for the GH27 domain of OsAPSE produced by a cell-free production system (CFPS), i.e., K_M_ = 0.67–4.68 mM and also the calculated V_max_ is higher compared to the CFPS experiment (i.e., V_max_ = 3.9–6.3 nM/s) but within the same order of magnitude in both cases [28]. The K_M_ value of other plant GH27 AGALs for the synthetic *p*NP-α-D-Gal*p* substrate is varying between 0.67–105 mM, with most reported K_M_ values being situated below 3 mM [49,50,51,52,53,54]. The calculated turnover number was k_cat_ = 0.15 s^−1^ for the used experimental setup and is within the order of magnitude of comparable enzymes. For instance, the GH27 AGAL from *Phaseolus vulgaris* was produced recombinantly in *E. coli* and yielded a k_cat_ in the range of 0.82–3.61 s^−1^, depending on the position of the nitrophenyl substituent being in ortho, meta or para position [55]. It is very likely that the high value for K_M_ is due to suboptimal protein folding, which is often observed in protein refolding experiments. Indeed, it was reported before that achieving the native protein structure upon refolding is often difficult and that the POI can get trapped in a stable but non-native state, with aberrant protein folding, and therefore also activity, which could explain the increased K_M_ [11,56,57]. Despite the divergent K_M_ value, we were still able to demonstrate and validate the AGAL activity of OsAPSE, as was also observed earlier in the CFPS experiment [28].

### 2.3. Expression in P. pastoris Yields Inactive Proteins of Interest

**Figure 2:**
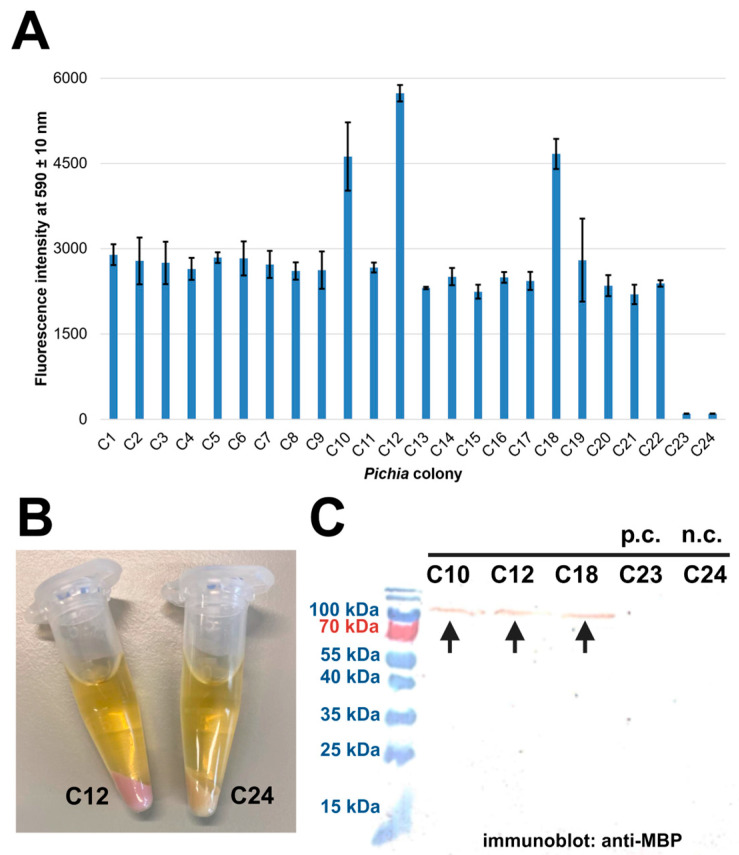
Expression of GH27_OsAPSE in *Pichia pastoris* X-33 cells. *Pichia* cultures containing the MBP-TEV-mCherry-GH27_OsAPSE-His_6_ fusion protein were analyzed for the presence of red fluorescence (**A**), which was obvious in centrifuged samples (**B**). The fusion proteins were detected with antibodies against the MBP tag (**C**) and are indicated with an arrow. The protein sizes of the mass ladder are mentioned in the same colors as in the blot. The positive control (p.c.) (C23) comprised a *Pichia* culture producing an antibody (without MBP/mCherry tag and is therefore not visible on the blot). The negative control (n.c.) (C24) included non-transformed *P. pastoris* X-33 cells (which also do not possess MBP-tagged proteins and are thus not visible on the blot). The error bars represent standard deviations from n = 3 technical replicates.

Although several attempts were undertaken to produce OsAPSE in the eukaryotic host *P. pastoris* (Table 1), most experiments did not yield production of the POI. However, the GH27 domain of OsAPSE, in combination with an *N*-terminal MBP tag, TEV protease site and mCherry RFP reporter tag, combined with a *C*-terminal His_6_ tag, was successfully produced (experiment 32). A total of 22 putatively transformed *Pichia* colonies were cultivated, and protein synthesis was induced. Afterwards, red fluorescence intensity was measured for every culture (Figure 2A) and was obvious in cultures C10, C12 and C18.

The presence of pink-colored proteins was even more obvious after centrifugation (Figure 2B) and is indicative for the production of the mCherry module, which was present in the MBP-TEV-mCherry-GH27_OsAPSE-His_6_ fusion protein (Figure 2C) and increased our screening efficiency. Unfortunately, the fusion protein made did not show measurable AGAL activity (Supplementary File S5.3). In contrast, an AGAL activity of 0.692 mOD/min was measured in the wild type negative control, which is attributed to background AGAL activity [58]. The inactivity of the GH27 domain may be explained by steric hinderance caused by either the MBP tag or the mCherry modules, which have a molecular weight of 40 kDa and 26.7 kDa, respectively. Attempts to remove the MBP tag using TEV protease cleavage were not successful, indicating that the MBP module is possibly also shielding the TEV cleavage site. Misfolding of the fusion protein is not likely, since the POI was produced in a eukaryotic background, promoting oxidative disulfide bridge formation. *P. pastoris* typically produces proteins with hyper-mannose *N*-glycans [20], which are considerably different compared to the native *N*-glycans of plant proteins. It was already demonstrated that the large hyper-mannose glycans can significantly affect the enzyme activity through steric hinderance of the catalytic site [59,60]. Protein production and purification experiments with the mutant GlycoDelete strain, which creates truncated and plant-like *N*-glycans [26], were however not successful (Table 2). An interesting research avenue to improve the proteolytic cleavage could be to co-produce a TEV protease in a parallel open reading frame, thereby ensuring in vivo cleavage of the recombinantly produced protein. This option has been explored before for expression of *A. thaliana* phytoglobins in *E. coli* [61]. Although it circumvents considerable costs of purchasing purified enzymes, it introduces a considerable cloning workload.

Interestingly, the recombinant production of AtAPSE (UniProt ID: F4JCI4), the *A. thaliana* homolog of OsAPSE, was successful in *P. pastoris* strain KM71 [29]. Despite bearing similar names, suggesting similar functions, there are some important differences between the two proteins. AtAPSE and OsAPSE share only 50.3% sequence identity and have strong structural differences, with an RMSD of 5.48 Å over 400 aligned residues (Supplementary File S6). The differences at the level of the sequence and protein structure can contribute to the many functional differences between the two homologues [28,29]. Indeed, sometimes a single point mutation is enough to jeopardize successful protein production, often when the point mutation is situated in a catalytic or structurally important site [62,63]. Furthermore, the differences between OsAPSE and AtAPSE can also explain why the success of AtAPSE production could not simply be transferred to OsAPSE. Nevertheless, there are examples that demonstrate how expression of homologues might be a solution [64]. However, it must be emphasized that this option is only feasible when larger protein families are being characterized and when there is the freedom to deviate from one POI. In contrast, if the research is focused on one specific POI, then the expression of a homolog is usually disadvantageous, as this is an approximation that introduces considerable uncertainty.

### 2.4. Expression in A. thaliana PSB-D Cell Cultures Results in Low Yields

**Figure 3:**
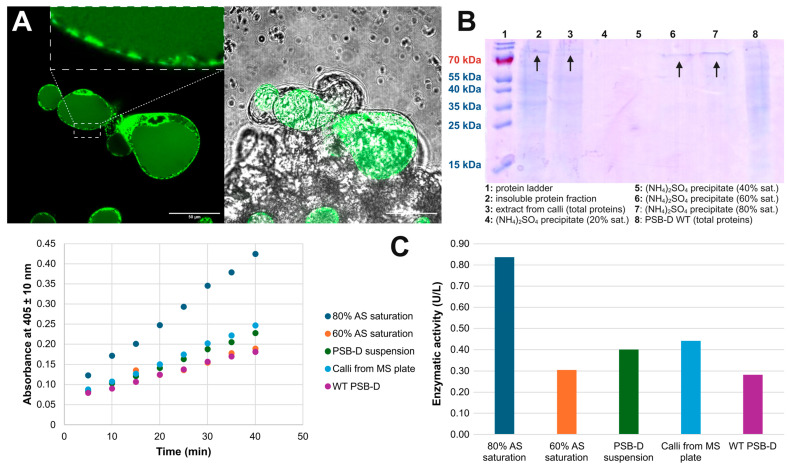
Production of OsAPSE in *Arabidopsis thaliana* PSB-D cell cultures. (**A**) Confocal fluorescence microscopy image of 600× magnified *A. thaliana* PSB-D cells co-expressing ER-localized EGFP with OsAPSE. *A. thaliana* PSB-D cells typically grow in clumps. The outlined box shows vesicular structures. The scale bar indicates a size of 50 µm. (**B**) Analysis of produced proteins in *A. thaliana* PSB-D cells by means of a Coomassie-stained SDS-PAGE gel. The protein of interest is shown with an arrow. The protein sizes of the mass ladder are mentioned in the same colors as in the blot. (**C**) Discontinuous enzymatic assays on different protein extracts from *A. thaliana* PSB-D cells transformed with pK7FWG2D::OsAPSE compared to non-transformed *A. thaliana* PSB-D cells. The observations in panel C were not repeated due to discontinuation of the *A. thaliana* PSB-D cultures.

*A. thaliana* PSB-D cell cultures were transformed with the pK7WG2D vector, co-producing the ER-localized EGFP, separately from the full-length OsAPSE protein or its individual subdomains (Table 1). Only the production of full-length OsAPSE was modestly successful (experiment 34). Green fluorescent signals were observed in the ER and in vesicular structures (Figure 3A), which is indicative for transcription and translation of ER-localized EGFP and OsAPSE. Since OsAPSE was produced with its native signal peptide, it was hypothesized that OsAPSE would be secreted to the extracellular medium. Therefore, the medium from the plant cell culture was collected, and extracellular proteins in the medium were precipitated. A small but distinct protein band around 70 kDa was observed for the medium at 60% (390 g/L) and 80% (561 g/L) (NH_4_)_2_SO_4_ saturation (Figure 3B). Subsequently, AGAL activity was measured during a discontinuous enzymatic assay (Supplementary File S5.4), making use of the crude, non-purified, precipitates. The highest AGAL activity was observed in the precipitate at 80% saturation, reaching an enzymatic activity of 0.80 U/L (Figure 3C), although we were unable to repeat the measurements due to discontinuation of the cell cultures. The transformed *A. thaliana* PSB-D cultures were discontinued for different reasons. The used MSMO medium contains high levels of sucrose and is therefore prone to bacterial and fungal contamination. More important, the expression of a transgene in plant cell cultures is typically limited in time as the expression of transgenes is (epi)genetically altered over successive generations and passages [65], until the point that the transgenic cells are outcompeted by non-transformed cells.

## 3. Discussion

Central to this study was OsAPSE, a multi-domain protein from rice, which is difficult to produce recombinantly. We explored a multitude of experimental conditions across several hosts, including different strains of *E. coli*, *P. pastoris* and cell suspension cultures of *A. thaliana*. The data of the CFPS experiments have been published in a recent publication [28]. Our results have shown that most well-established interventions did not improve the solubility of the POI, including optimization of the expression conditions, codon optimization, usage of specialized strains capable of synthesizing disulfide bridges aiming for approximating the native protein structure, design of mutational variants and chaperone co-expression. POI solubility was improved by using solubility tags such as MBP or when protein refolding was considered. The MBP-tagged POI was soluble, but enzymatically inactive, most probably due to steric hinderance caused by the size of the tag. The refolded TRX-OsAPSE protein, however, demonstrated clear AGAL activity, which led to confirmation of the kinetic parameters K_M_ and V_max_, which were in line with the earlier observed values [28]. Most successes were obtained when the POI was produced in *A. thaliana* PSB-D cells, a eukaryotic (plant cell) host. This observation reinforces the widely accepted principle that production of a POI occurs most optimal when the origin of the host and the sequence to be expressed are related—the closer the better.

Our results reinforce the highly empirical nature of recombinant protein production, where even well-established optimization strategies may fail, depending on the nature of the POI. Despite testing various conditions, OsAPSE production remained challenging, illustrating the trial-and-error nature of recombinant protein production. Our results also emphasize the importance of ‘negative data’ in protein research, as these can guide future optimization efforts and methodological refinements. Our findings provide practical insight for researchers facing similar challenges, underscoring the need for systematic screening, flexible experimental design and a realistic approach to optimizing recombinant protein production. Therefore, when confronted with failing recombinant protein production of protein insolubility, we recommend following considerations:

1. **First, it is recommended to optimize the operational production parameters** such as the incubation temperature, shaking speed for aeration of the cultures, incubation time, concentration of inducer molecule for transcript expression, medium composition (*f.i.*, presence of solubility enhancing additives, considering auto-induction medium), culture volume and cell lysis method [8,66]. In practice, it is advised to reduce the temperature during the induction phase because this reduces the protein biosynthesis rate and increases the chance of obtaining a soluble POI.
2. **Second, reconsideration of the expression construct may be advised**. Researchers should take codon bias into account and optimize/harmonize the coding sequence. Several online tools make adjustments to the amino acid sequence, including deep learning and artificial intelligence, are available [39,40,67]. To circumvent issues with solubility, it might be considered to mutate hydrophobic residues to hydrophilic residues, thereby increasing the hydrophilicity of the POI and increasing the chance of obtaining soluble POI. This specific approach was successful for the production of Interleukin-2, after several point mutations [68,69]. However, the targeted residues should be chosen carefully, as catalytic pockets or ligand binding sites often involve hydrophobic residues, for instance in the case of carbohydrate-binding proteins. In addition, if non-optimized sequences are used in a prokaryotic system such as *E. coli*, the Rosetta^®^ strain can be considered. This strain is engineered with additional transfer-RNAs for enhancing translation of eukaryotic proteins with ‘rare codons’ [70]. Next to codon bias, the addition of solubility tags may be considered. Widely used solubility tags include MBP, GST and TRX [71]. Successful protein production is, however, not guaranteed when employing solubility tags. Several parameters can exert an effect on the solubility of the new fusion protein [72,73], for instance the positioning (*C*- or *N*-terminal) of the solubility tag, the size of the tag, the number of tags … It should be taken into account that fusion with a large solubility tag may affect protein activity by sterically shielding active states and introducing the need for proteolytic removal of the solubility tag. If a TEV site is used, the simultaneous production of a TEV protease can be considered allowing in vivo proteolytic cleavage, thereby avoiding the need for purchasing expensive commercial enzymes and simplifying the downstream purification procedure. Finally, selection of a proper solubility tag and positioning towards the protein domain of interest often needs to be established and/or optimized empirically.
3. **Another option for prokaryotic protein production is to use modified host strains**. Several modified hosts are available that may accommodate the researchers’ individual needs and are often equipped with additional chaperones. These chaperones are able to recognize unproperly folded proteins and prevent them from aggregation. Typical chaperons include the heat-shock proteins and have been engineered in strains to circumvent issues with protein aggregation [74], and may assist in proper protein folding [75]. The *E. coli* ArcticExpress^®^ strain coproduces the Cpn10 and Cpn60 chaperonins from *Oleispira antarctica*, allowing protein production at lowered temperatures (4–10 °C), potentially accommodating a lower protein biosynthesis rate and therefore limiting the risk of protein aggregation and IB formation [76]. Another example is the *E. coli* SHuffle^®^ strain, which is equipped with the disulfide bond isomerase chaperone, allowing formation of disulfide bridges in the cytosol [42], hereby increasing solubility of proteins that require disulfide bridges [77]. The engineered GlycoDelete strain of *P. pastoris*, allows recombinant protein production in the absence of hyper-glycosylation [25];
4. Next to usage of engineered host strains, **it can be considered to co-express molecular chaperones that are situated upstream of downstream from the native gene of interest**. There is sufficient evidence that these chaperones, mostly heat-shock proteins, are co-expressed under native conditions to ensure proper POI folding [78].
5. **A frequently utilized approach is to produce the POI in IBs and perform subsequent protein unfolding and refolding** [16]. Protein refolding is controversial since the refolding step does not always restore the native folding; it might trap the protein in a non-native state resulting in an inactive protein. Protein refolding protocols require extensive optimization and are highly empirical [79,80,81]. Nevertheless, the performance of refolding strategies has been demonstrated many times before [14,15];
6. **Changing the expression host may be considered**, since the success of recombinant protein production is for a large part determined by the host used. A study producing 29 human proteins in *E. coli* and *P. pastoris* demonstrated that all of the POI were soluble when using *P. pastoris*, compared to only 31% when using *E. coli* [82]. Eventually, CFPS or phage/yeast display may be opted when traditional cell-based strategies are not successful [83]. CFPSs make use of cell lysates and contain all the necessary components for protein synthesis. Both prokaryotic CFPS (*f.i.*, cell lysates of *E. coli*, archaeans) and eukaryotic CFPS (*f.i.*, tobacco Bright Yellow-2 lysates, rabbit reticulocyte lysates) systems exist, but similar to conventional recombinant protein production, the CFPS should be chosen carefully, taking into account the same considerations as mentioned above. However, CFPS may be confronted with reduced yields [84,85]. Phage/yeast display has the advantage that the POI is produced by the host and presented at the cell surface, thereby removing the need for tedious or laborious optimization of protein production and purification. However, yeast/phage display may be confronted with similar issues as with traditional recombinant protein production, since the same constraints regarding non-native expression remain valid;
7. **A final option is considering to produce a homolog of the POI**, as it was shown before that the success of recombinant protein production may vary between homologues [64]. It should be kept in mind that this research avenue is especially suitable when exploring, *f.i.*, enzyme families or other cases where researchers are not bound to one particular POI.

## 4. Materials and Methods

### 4.1. Construct Design, Gene Cloning and Host Transformation

The coding sequence of OsAPSE (Supplementary File S7) was codon-optimized for expression in different hosts (Table 1) and synthesized using the GeneArt Gene Synthesis service (Thermo Fisher Scientific, Waltham, MA, USA). Prior removal of the native signal peptide, addition of restriction sites and stop codons were performed when necessary. The Codon Harmonizer of the University of Graz, Austria was employed (http://biocatalysis.uni-graz.at/sites/codonharmonizer.html; accessed on 16 April 2025). For codon optimization, 4 algorithms were used from different websites: Integrated DNA Technologies (https://eu.idtdna.com/CodonOpt; accessed on 16 April 2025), EUROFINS (https://eurofinsgenomics.eu/; accessed on 16 April 2025), VectorBuilder (https://en.vectorbuilder.com/tool/codon-optimization.html; accessed on 16 April 2025) and NovoPro (https://www.novoprolabs.com/tools/codon-optimization; accessed on 16 April 2025). Codon bias was studied by using the Biologics International Corporation Rare Codon Analyzer tool [86] and the Rare Codon Caltor (https://people.mbi.ucla.edu/sumchan/caltor.html; accessed on 16 April 2025). The Protein Repair One-Stop Shop (PROSS) tool (https://pross.weizmann.ac.il/step/pross-terms/; accessed on 16 April 2025) was used to generate mutational variants of OsAPSE. The effect of the mutations on the resulting protein structure was assessed in PyMol v2.5.4 using calculation of root-mean square deviation (RMSD) values [87,88].

The codon-optimized sequence of OsAPSE or its subdomains was subcloned in the pJET1.2 shuttle vector (Thermo Fisher Scientific) and transformed in chemically competent *E. coli* Top10 cells (Thermo Fisher Scientific) by applying a heat-shock (42 °C, 42 s) and subsequent incubation on ice in the presence of lysogeny broth (LB) (Duchefa Biochemie, The Netherlands). Upon verification of the construct by means of colony PCR, plasmid DNA was isolated using the GeneJET^TM^ Plasmid Miniprep kit (Thermo Fisher Scientific) and verified by means of gene sequencing by LGC Genomics GmbH (Berlin, Germany) using vector-specific primers (Supplementary File S8). Depending on the intended expression construct and expression host, different methods for gene cloning and host transformation were utilized (Table 1).

#### 4.1.1. Gene Cloning for Expression in *E. coli*

Most constructs for expression in *E. coli* made use of inducible pET-vectors under influence of the T7 system (Table 1), using restriction and ligation as cloning methods. Restriction enzymes were purchased at New England Biolabs (Ipswich, MA, USA). The insert sequences in the pJET1.2 shuttle vector were cloned in the expression vector by means of a double digest using 5 µg of each vector (in separate reactions) and 2.5 U of each restriction enzyme in combination with 10× rCutSmart buffer, for 1 h at the required temperature, followed by an inactivation at 80 °C for 20 min. The expression vector and insert sequence were purified using the QIAquick^®^ PCR & Gel Cleanup Kit (Qiagen, Hilden, Germany). The inserts were ligated into the expression vectors in a 3/1 insert-to-vector ratio using 5 U T4 DNA ligase (Thermo Fisher Scientific) and 10× ligase buffer (Thermo Fisher Scientific). The resulting recombinant expression vectors were transformed in *E. coli* Top10 cells and verified by means of colony PCR and gene sequencing, as described earlier. Transformed Top10 cells were propagated in selective LB for plasmid extraction and transformation of the expression strain by means of heat-shock transformation. Verified expression strains were immediately used for recombinant protein production, as described in Section 4.2.1.

The specific pDEST and pVTD expression vectors were obtained by using Gibson assembly followed by Golden/Green Gate cloning or the related VersaTile technology. Sequences were prepared for Gibson assembly of the entry vector based on so-called position markers that allow seamless and modular cloning. More details concerning the used protocols are covered in [89,90]. After Gibson assembly, the resulting entry clones were transformed in *E. coli* Top10 cells and verified as mentioned earlier. The destination vector was assembled by adding the requested modules (*f.i.*, purification tags, linkers, coding sequences) with predefined position markers in equimolar amounts and in the presence of type-II restriction enzymes (New England Biolabs) [89,90]. The resulting destination vectors were transformed in *E. coli* Top10 cells and verified by means of colony PCR and gene sequencing, as described earlier. Transformed Top10 cells were propagated in selective LB for plasmid extraction and transformation of the expression strain by means of heat-shock transformation. Verified expression strains were immediately used for recombinant protein production, as described in Section 4.2.1.

#### 4.1.2. Gene Cloning for Expression in *P. pastoris*

All expression experiments in *P. pastoris* made use of the methanol-inducible pPICZαA expression plasmid, under control of an alcohol oxidase 1 promotor. Similarly to the expression experiment in *E. coli*, two different cloning methods were used, mainly depending on the used expression vector. Both pPICZαA and a modified variant were used. The codon-optimized sequence of OsAPSE for expression in *P. pastoris* strains, present in the pJET1.2 shuttle vector, was cloned in the original pPICZαA plasmid using restriction and ligation. All plasmids were submitted to a double digest as described above. After verification, propagation and isolation, 5 µg expression vector was linearized using 1 U PmeI restriction enzyme (New England Biolabs) combined with 10× rCutSmart Buffer at 37 °C for 1 h. The restriction enzyme was inactivated at 65 °C for 30 min and immediately used for transformation of *P. pastoris* strains. The Green Gate-compatible pPICZαA expression vector was assembled in exactly the same way as described in the previous section.

Aliquots of 80 µL electrocompetent *P. pastoris* strains were thawed on ice and carefully pipetted in pre-chilled 1 cm-electroporation cuvettes. Around 100 ng of linearized expression vector was added to the electroporation cuvette and transformed into *P. pastoris* by means of an electric pulse, delivered by a MicroPulser Electroporator (Bio-Rad, Hercules, CA, USA) using the built-in settings for *P. pastoris*. Immediately after the electric pulse, 1 mL of sterile and ice-cold sorbitol (Chem-Lab, Zedelgem, Belgium) at a concentration of 1 M was added and the transformation mixture was incubated at 28 °C for 2 h while shaking at 250 rpm on a rotary shaker. Afterwards, the transformation mixture was plated out on selective YPD plates (10 g/L yeast extract, 20 g/L peptone, 20 g/L glucose) and incubated for 2–3 days at 28 °C in darkness. The resulting fungal colonies are to be used in the protein production screening experiment as outlined in Section 4.2.2.

#### 4.1.3. Gene Cloning for Expression in *A. thaliana* PSB-D Cell Cultures

The experiments making use of *A. thaliana* PSB-D cell cultures all made use of the Gateway-compatible pK7WG2D vectors, which are constitutive expression vectors under influence of the 35S promotor [91]. The codon-optimized sequence for expression in *A. thaliana*, present in the pJET1.2 shuttle vector, was used in consecutive PCR reactions (Supplementary File S8) to provide the coding sequences with attB sites, which allows Gateway cloning. In between steps, the intermediate sequences were subcloned in the pJET1.2 shuttle vector and verified by colony PCR and gene sequencing, as described above. The BP and LR reactions for homologous recombination were initiated in the presence of 1X TE buffer (10 mM Tris-HCl pH 8, 1 mM Na_2_EDTA pH 8), 100–200 ng/µL insert material (i.e., either the attB-flanked coding sequence for the BP reaction, or the entry clone for the LR reaction) and 2 U of BP Clonase^TM^ or LR Clonase^TM^ enzyme mix (Thermo Fisher Scientific). The reaction mixtures were incubated overnight at room temperature. The day after, the recombination reactions were incubated with 1 µL proteinase K for 10 min at 37 °C to inhibit the recombination reaction.

The verified destination vector was immediately used for transformation of *Agrobacterium tumefaciens* strain EHA105. Aliquots of 80 µL electrocompetent *A. tumefacience* cells were thawed on ice and carefully pipetted in pre-chilled 1 cm-electroporation cuvettes. Around 100 ng of destination vector was added to the electroporation cuvette and transformed into *A. tumefaciens* by means of an electric pulse, delivered by a MicroPulser Electroporator (Bio-Rad) using the built-in settings for *A. tumefaciens*. Immediately after the electric pulse, 900 µL yeast extract broth (5 g/L beef extract, 5 g/L peptone, 1 g/L yeast extract, 5 g/L sucrose) was added and the bacteria were incubated at 28 °C for 2 h while shaking at 200 rpm. Afterwards, the cells were plated out on selective plates and incubated for 2 days in darkness at 28 °C. Putatively transformed *Agrobacterium* cells were selected by means of colony PCR and kept in culture in parallel.

The PSB-D cell suspension culture of *Arabidopsis thaliana* (L.) Heynh. Ecotype Landsberg *erecta* were kindly provided by prof. Geert De Jaeger (Vlaams Instituut voor Biotechnologie, Plant Systems Biology) and transformed according to [92]. In short, 200 µL of a recombinant *Agrobacterium* culture at optical density at 600 nm (OD_600_) = 1 was co-cultivated with 3 mL of a 7-day-old PSB-D culture at 25 °C for 2 days, with gentle agitation at 130 rpm. Afterwards, the transformation mixture was sub-cultivated in selective Murashige and Skoog medium with Minimal Organics (MSMO) medium for 7 days. From that point, the suspension culture was maintained weekly by subculturing 7 mL of a 7-day-old culture into 45 mL of selective MSMO in a 100 mL micropore-sealed Erlenmeyer, with permanent incubation at 25 °C and shaking at 130 rpm in darkness. Proteins were extracted as described in Section 4.2.3.

### 4.2. Protein Production and Extraction

#### 4.2.1. *Escherichia coli*

Overnight cultures (5 mL), with sterile selective LB, were inoculated with a single colony and incubated at 37 °C for 16–18 h while shaking at 220 rpm. The overnight cultures were sub-cultured in sterile LB (upscaling in 1/20 ratio of overnight culture to sterile LB) and incubated at 37 °C. the OD_600_ was measured regularly. When OD_600_ = 0.6–0.8, the culture was divided into several flasks, and protein production was induced by adding isopropyl β-D-1-thiogalactopyranoside (Chem-Lab) to a final concentration of 0.5–1 mM. In the case of *E. coli* BL21-AI, arabinose (Thermo Fisher Scientific) was added to a final concentration of 0.2% (*w*/*v*). The cultures were incubated at different temperatures (37 °C, 28 °C, 21 °C, 16 °C or 4 °C), depending on the used strain, for 20–24 h while shaking at 220 rpm. When the pET-22b(+) plasmid was used, POIs were produced with an *N*-terminal *pel*B signal sequence, supposedly guiding the produced proteins to the periplasm and medium. Medium protein fractions were isolated by means of precipitation with 100% trichloroacetic acid (TCA) (Carl-Roth), rinsing with ice cold acetone and restitution of the pellet in 50 µL non-buffered Tris. Proteins present in the periplasm were collected by administering an osmotic shock by incubating the cell pellet in a TSE buffer containing 50 mM Tris-HCl pH 7.5, 30% (*w*/*v*) sucrose (Sigma-Aldrich) and 10 mM EDTA (Carl-Roth), while stirring gently for 30 min. The cells are collected through centrifugation (30 min, 15,000× *g*, 4 °C) and resuspended in ice-cold 5 mM MgSO_4_ (Chem-Lab) and incubated in an ice bath while shaking gently. During the shaking step, the proteins are released from the periplasmic fraction. The released periplasmic proteins are then collected by TCA precipitation, acetone rinsing and restitution in non-buffered Tris.

Throughout this study, multiple methods for protein extraction were utilized. Mostly, cell lysis was achieved through mechanical rupture using a sonicator (amplitude 30%, total sonication time of 15 min, on ice), or using glass beads (ø 0.2–0.5 mm) (Carl Roth, Karlsruhe, Germany), combined with chemical lysis using an extraction buffer: either the NEB-Express^®^ *E. coli* Lysis Reagent (New England Biolabs) or the TGH1 (i.e., Triton-Glycerol-HEPES) aqueous extraction buffer containing 1% (*w*/*v*) Triton X-100 (Sigma-Aldrich, Saint-Louis, MO, USA), 10% glycerol (Chem-Lab) and 50 mM HEPES (Santa Cruz Biotechnology, Dallas, TX, USA), 300 mM NaCl (Chem-Lab), supplemented with 2 tablets of EDTA-free cOmplete^TM^ Protease Inhibitor (Hoffmann-La Roche, Basel, Switzerland) per 100 mL of extraction buffer and 0.1 mM phenylmethyl-sulfonyl fluoride (PMSF) (Thermo Fisher Scientific). Extractions were executed at room temperature for 20–30 min. Afterwards, the soluble and insoluble fractions were separated by centrifugation for 30 min at 4000× *g* (4 °C). The soluble cytoplasmic protein fraction is then present in the supernatant, and the insoluble cytoplasmic protein fraction resides in the pellet.

#### 4.2.2. *Pichia pastoris*

Experiments using *P. pastoris* strains were limited to small-scale screenings in deep-well plates (24 wells, 2 mL per well) and did not proceed to the upscaling stage. Transformed *P. pastoris* cells bearing methanol-inducible expression plasmids were resuspended in selective BMGY medium containing 1 g/L yeast extract (Merck, Darmstadt, Germany), 2 g/L peptone (Merck), 1.34% (*w*/*v*) yeast nitrogen bae without amino acids but with (NH_4_)_2_SO_4_ (Chem-Lab), 100 mM KH_2_PO_4_/K_2_HPO_4_ (Merck) buffer at pH 6 and 10% (*w*/*v*) glycerol. The *Pichia* cultures were incubated at 28–30 °C for 3 days in darkness, sealed with Millipore tape, while shaking at 240 rpm for adequate aeration. Afterwards, the cells were washed with BMGY and resuspended in BMMY medium, which contains 10% (*v*/*v*) sterile methanol (Chem-Lab), instead of glycerol. The following 2 days, the *Pichia* cultures were periodically spiked with methanol to a final concentration of 1% (*v*/*v*). For the constructs co-expressing an mCherry RFP reporter, protein production was screened by investigating red fluorescence intensity. This was performed by analyzing 50 µL of *Pichia* culture in 96-well plates using a TECAN Infinite 200 PRO (TECAN, Männedorf, Switzerland) plate reader. RFP was exited at 560 ± 20 nm and emitted light was detected at 590 ± 10 nm. *Pichia* cultures showing high red fluorescence intensities were considered for further analysis and harvested by means of centrifugation. Extracellular proteins, secreted to the medium (total volume of 2 mL), were collected through acid precipitation using 100% TCA and washing with 100% ice-cold acetone (Chem-Lab). The protein pellet was collected by centrifugation (10 min, 10,000× *g*, 4 °C), after which it was dried to the air and dissolved in 50 µL of 1 M non-buffered Tris (MP Biomedicals, Irvine, CA, USA). For the intracellular proteins, the cultures (2 mL volume) were harvested through centrifugation (10 min, 10,000× *g*, room temperature) and dissolved in TGH1 extraction buffer combined with glass beads (ø 0.2–0.5 mm), vigorous vortexing and cooling on ice, to achieve cell lysis. The lysates were centrifuged (30 min, 10,000× *g*, 4 °C) and the intracellular proteins were present in the supernatant. The pellet contained mainly cell debris and insoluble proteins.

#### 4.2.3. *Arabidopsis thaliana* PSB-D Cell Cultures

Continuous cultures of *A. thaliana* PSB-D cells were maintained and sub-cultured weekly in fresh selective MSMO [92]. Simultaneously, 2 mL of the cell suspension was added to fresh selective MSMO plates and incubated at 21 °C in darkness, as a back-up for failing or contaminated cultures. We made use of the pK7WG2D expression vector, which contains an endoplasmic reticulum (ER)-localized version of EGFP as reporter protein [91]. For each passage of the cell cultures, the presence of green fluorescence was assessed through confocal microscopy [93] using a Nikon A1R confocal laser scanning microscope (Nikon Instruments, Tokyo, Japan) and a CFI Plan Apo VC 60x WI DIC (NA1.2) objective. GFP was excited using the 488 nm argon laser line and detected using an 515–530 nm emission filter. Images were processed using the ImageJ v1.54 software [94]. All original microscopy raw data is included in Supplementary File S9 and the processed images in Supplementary File S10.

Cells from a one-week old culture (or two-week old calli from MS plates) were harvested and crushed to a fine cell powder using liquid nitrogen, a mortar and pestle. Approximately 0.5–1.0 g of cell powder was combined with 0.5–1.0 mL extraction buffer (*f.i.*, TGH1) and incubated for 20–30 min at room temperature, after which the cell debris was separated from the protein solution through centrifugation (30 min, 4000× *g*, 4 °C). The soluble intracellular protein fraction was collected in the supernatant, whereas the pellet contains the insoluble protein fraction. Thereafter, the supernatant was submitted to (NH_4_)_2_SO_4_ (Chem-Lab) precipitation at 80% saturation (561 g/L) to precipitate the secreted protein fraction for 3–4 days at 4 °C. NaN_3_ (Sigma-Aldrich) to a final concentration of 0.1–0.5% (*w*/*v*) was added to prevent microbial growth in the protein solution. Protein precipitates were harvested through centrifugation (30 min, 4000× *g*, 4 °C) and dissolved in 1–2 mL of 50 mM Tris-HCl pH 7.5.

### 4.3. Protein Analysis

#### 4.3.1. Protein Concentration

Protein concentrations were determined using either the colorimetric Bradford assay [95] at 595 nm or UV spectrophotometry at 280 nm. Bovine serum albumin (BSA) was used as a reference protein in standard curves between 0–1 mg/mL.

#### 4.3.2. SDS-PAGE and Western Blot

Discontinuous acrylamide gels with 0.1% SDS (MP Biomedicals) were prepared, containing 4% acrylamide in the stacking gel (pH 6.8) and 15% in the separating gel (pH 8.8). Protein samples were incubated at 98 °C for 10 min in 4× sample buffer containing 1 M Tris-HCl pH 6.8, 8% (*w*/*v*) SDS, 40% (*w*/*v*) glycerol, 0.4% (*w*/*v*) bromophenol blue and 1.125 M beta-mercaptoethanol (Carl Roth), prior to electrophoretic analysis in a continuous electric field (180–200 V) for 1 h. Running buffer containing 25 mM Tris, 200 mM glycine (Merck) and 0.1% (*w*/*v*) SDS was used. Gels were stained with acidic 0.1% (*w*/*v*) Coomassie Brilliant Blue R250 (Merck) and destained with destaining solution containing 2.5 M technical ethanol (Chem-Lab) and 1.3 M glacial acetic acid (Chem-Lab).

After SDS-PAGE, proteins were blotted on methanol-activated PVDF membranes (GE Healthcare, Chicago, IL, USA) by semi-dry electroblotting (Bio-Rad) in Towbin buffer containing 25 mM Tris, 20% (*v*/*v*) methanol and 192 mM glycine. Membranes were incubated in 5% (*w*/*v*) milk powder solution (AppliChem GmbH, Darmstadt, Germany). His_6_-tagged proteins were detected using consecutively 1/5000 THE^TM^ His-tag monoclonal antibody (GenScript, Piscataway, NJ, USA), 1/1000 polyclonal rabbit anti-mouse antibody conjugated with horseradish peroxidase (Agilent/DAKO, Santa Clara, CA, USA), 1/300 peroxidase anti-peroxidase antibody (Sigma-Aldrich). All antibodies were incubated for 1 h, except the peroxidase anti-peroxidase, which was incubated for 45 min. For detection, 100 mM Tris-HCl pH 7.6 containing 1 mM 3,3′-diaminobenzidine (Thermo Fisher Scientific) containing 320 µM H_2_O_2_ (Acros Organics, Geel, Belgium) was used. Trissaline containing 10 mM Tris, 150 mM NaCl and 0.1% (*v*/*v*) Triton X-100 was used as a diluent for all antibodies and for membrane washes (3 × 5 min) in between antibody incubations.

All original SDS-PAGE gels and blots are included in Supplementary File S10.

### 4.4. Downstream Analyses

#### 4.4.1. Protein Refolding

Protein refolding was executed after recombinant protein production of certain expression constructs in *E. coli* (Table 1). Cells from a 2 L bacterial culture were harvested and resuspended in 50 mL sonication buffer (50 mM Tris, 100 mM NaCl, 1 mM PMSF, pH 8). The suspension was placed in ice water for temperature control and sonicated at 30% amplitude in pulses of 5 s, alternating with 5 s of pause (QSonica LLC, Newton, CT, USA), for a total sonication time of 15 min. After sonication, the IBs were isolated by means of centrifugation (30 min, 15,000× rpm, 4 °C). The IBs were thoroughly washed and vortexed, twice, with washing buffer 1 (i.e., extraction buffer supplemented with 0.1% (*v*/*v*) Triton X-100). Finally, the IBs were washed with washing buffer 2 (i.e., extraction buffer without PMSF) and collected through centrifugation (30 min, 15,000 rpm, 4 °C). The IBs were then solubilized by using a solubilization buffer containing 20 mM NaH_2_PO_4_, 500 mM NaCl, 5 mM beta-mercaptoethanol, 6 M guanidine hydrochloride (MP Biomedicals), 5 mM imidazole (Merck) at pH 7.5. Thereafter, the solubilized IBs were purified by nickel affinity chromatography, using column buffer 1 containing 20 mM Na_2_HPO_4_, 500 mM NaCl, 5 mM beta-mercaptoethanol, 8 M urea (Merck), 20 mM imidazole at pH 7.5, for column equilibration and washing. Column buffer 2 (i.e., column buffer 1 supplemented with 300 mM imidazole), was used for elution of the unfolded proteins. Finding the optimal refolding conditions needs to be determined empirically, as there is no universal refolding buffer. Usually, a combination of a buffer system (*f.i.*, PBS, Tris, HEPES, MES, citrate buffer, piperazine buffer, …) at a pH between 4–10, supplemented with combinations of additives (*f.i.*, salts, metal ion chelators, polyols, reducing agents, detergents, polymers, chaotropic agents, amino acids and monosaccharides), is considered [16,79,80,81,96,97]. Semi high-throughput screening methods have been developed in the past and have been proven useful for reference proteins including lysozyme, carbonic anhydrase B and glutamate receptor R2 [79,80,81]. Refolding conditions leading to soluble or insoluble proteins were evaluated by measuring the turbidity of the mixtures spectrophotometrically in the range of 300–400 nm. Low turbidity values (A < 0.05) indicate that the refolding buffer is a good solvent for the POI and does not cause insolubility and aggregation. We used different combinations of buffers and additives, as used in [80,81], similarly to [97], using 10 µL of purified, unfolded proteins, combined with 190 µL of refolding buffer, and measured the turbidity at 405 ± 10 nm after overnight incubation at room temperature. The success of the refolding is usually confirmed through dynamic light scattering or circular dichroism, but can also be confirmed through other downstream analyses, such as enzymatic activity analysis (Section 4.4.2), since activity is determined by the extent to which the native protein structure has been restored [98].

#### 4.4.2. Enzymatic Activity Assays

The success of protein refolding after protein production in *E. coli*, or when soluble proteins were obtained in other production hosts (Table 1), was assessed by performing enzymatic activity tests. Activity tests made use of the synthetic substrate *p*-4-nitrophenol-α-D-Galactopyranoside (*p*NP-α-D-Gal*p*) (Thermo Fisher Scientific) at a final concentration of 25 mM to screen for AGAL activity. The enzyme solution at a final working concentration of 50 µg/mL (i.e., 66 nM) was used in 50 mM Tris-HCl pH 8 buffer. Discontinuous enzymatic assays at a temperature of 25 °C were set up. Samples of 100 µL were taken every 5 min and inactivated in 100 µL of 0.2 M Na_2_CO_3_ (pH 11). Afterwards, absorbance measurements at 405 ± 10 nm were executed. Kinetic parameters were calculated by using Eadie–Hofstee [99] and Hanes–Woolf [100] linearization methods.

## 5. Conclusions

This study exemplifies the difficulties researchers may face when expressing eukaryotic sequences, especially in non-native host systems and highlights important considerations and solubility-enhancing strategies that might resolve the issue of protein insolubility. While *E. coli* is the most commonly used production host in research and industry [101], its limitations—particularly protein insolubility and improper folding—are major bottlenecks. The most important rule of thumb remains to ‘know your protein’ and adapt the expression conditions to meet the requirements of the POI with respect to structural characteristics and PTMs [24]. The experimental design options are endless, and every experimental parameter comes with its own advantages and limitations, requiring careful consideration [9].

## Figures and Tables

**Table 1:** Overview of the executed recombinant protein production experiments in this study.

EXP	Host Organism	Strain or Type	Expression Plasmid	CDS *^a^*	CDS Adjustments	Tags	Cloning Method	Lysis Method	Result	Refolding
1	*E. coli*	BL21	pET-22b(+)	OsAPSE	Codon Opt.	*N*-pelB+*C*-His_6_	Res. & Lig	LyB	Ins.	Yes
2		BL21-AI							Ins.	No
3		pLysS							Ins.	No
4		Rosetta							Ins.	No
5		ArcticExpress							Ins.	No
6		SHuffle							N.P.	No
7				GH27					Ins.	No
8	*E. coli*	BL21	pET-28a(+)	OsAPSE	Codon Opt.	*C*-His_6_	Res. & Lig	LyB	Ins.	No
9		ArcticExpress							Ins.	No
10	*E. coli*	BL21	pVTD13	OsAPSE	Codon Opt.	*N*-GST+*C*-His_6_	VersaTile cloning	LyB	N.P.	No
11				GH27					N.P.	No
12				ricin-B					Ins.	No
13				GH-all-beta					Ins.	No
14	*E. coli*	BL21 Star	pET-32a(+) *^b^*	OsAPSE	Codon Opt.	*N*-TRX+*C*-His_6_	Res. & Lig	Son.	Ins.	Yes
15		ArcticExpress							Ins.	No
16		Rosetta							Ins.	No
17	*E. coli*	BL21	pET-21a(+)	OsAPSE	Harm. *^c^*	*C*-His_6_	Res. & Lig	LyB	Ins.	No
18		Rosetta							N.P.	No
19		BL21			Codon Opt. *^d^*				Ins.	No
20		SHuffle							N.P.	No
21		BL21			PROSS *^e^*				Ins.	No
22		SHuffle							N.P.	No
23	*E. coli*	BL21	pDEST	OsAPSE	Codon Opt.	*N*-MBP+TEV site+*C*-FLAG_3_ or *C*-HA_3_ or *C*-His_6_	GG cloning	LyB	Ins.	No
24				ricin-B					Ins.	No
25				GH-β					Ins.	No
26				GH27				LyB + Son.	Soluble	No
27						*N*/*C*-MBP_2_+*C*-His_6_		LyB	Ins.	No
28						*N*-GST+*C*-His_6_			Ins.	No
29	*P. pastoris*	X-33	pPICZαA	OsAPSE	Codon Opt.	*N*-α-factor+*C*-His_6_	Res. & Lig	Beads + LyB	N.P.	No
30		GlycoDelete							N.P.	No
31		KM71H							N.P.	No
32	*P. pastoris*	X-33	Modified pPICZαA	GH27	Codon Opt.	*N*-MBP+*C*-RFP	GG cloning	Beads + LyB	Soluble	No
33						*N*/*C*-MBP_2_+*C*-His_6_			N.P.	No
34	*A. thaliana*	PSB-D cell culture	pK7WG2D	OsAPSE	Codon Opt.	Reporter EGFP+*C*-His_6_	GW cloning	Cryo + LyB	Soluble	No
35				GH27					N.P.	No
36				ricin-B					N.P.	No
37				GH-β					N.P.	No
38	BY-2 CFPS	ALiCE	pALiCE02	GH27	Codon Opt.	*C*-His_6_	Res. & Lig	None	Soluble *^f^*	No

Abbreviations: CDS (coding sequence), CFPS (cell-free production system), Cryo (cryogenic crushing), EXP (experiment), GG (Golden/Green Gate), GW (Gateway), Harm. (harmonization), Ins. (insoluble), LyB (lysis buffer), N.P. (not produced), Opt. (optimization), Res. & Lig. (restriction and ligation), Son. (sonication), TEV (Tobacco Etch Virus). Remarks: The terminal positions of tags are indicated with *N*’ and *C*’. Notes: *^a^* OsAPSE refers to the full-length coding sequence, while GH27, ricin-B and GH-β refer to the subdomains of OsAPSE; *^b^* outsourced to GenScript; *^c^* 2 harmonization variants were used; *^d^* 4 optimization algorithms available on IDT, EUROFINS, VectorBuilder and NovoPro were utilized; *^e^* Protein Repair One-Stop Shop; *^f^* not part of this study but recently published [28].

**Table 2:** Overview of the PROSS variants of OsAPSE and their solubility when expressed.

	WT	Variant 1	Variant 2	Variant 3	Variant 4	Variant 5	Variant 6	Variant 7	Variant 8	Variant 9
Sequence identity compared to OsAPSE	100	98.7	97.8	97.7	96.5	95.7	95.5	94.6	93.5	92.8
Number of mutated amino acid residues	0	8	14	15	23	28	29	35	42	47
RMSD (Å) compared to OsAPSE	0	0.0684	0.0825	0.0881	0.0908	0.1017	0.1030	0.0963	0.1081	0.1113
POI produced recombinantly?	Yes	Yes	No	Yes	No	Yes	No	Yes	No	No
Soluble POI?	No	No	No	No	No	No	No	No	No	No

## Data Availability

The original contributions presented in this study are included in the article and Supplementary Materials. Further inquiries can be directed to the corresponding author.

## Supplementary Materials

The following supporting information can be downloaded at: https://www.mdpi.com/article/10.3390/ijms26188974/s1.

## Abbreviations

The following abbreviations are used in this manuscript:

AGAL	α-D-Galactopyranosidase
BSA	Bovine Serum Albumin
CAI	Codon Adaptation Index
CFPS	Cell-Free Production System
EGFP	Enhanced Green Fluorescent Protein
ER	Endoplasmic Reticulum
GST	Glutathione S-Transferase
IB	Inclusion Body
LB	Lysogeny Broth
MBP	Maltose-Binding Protein
MSMO	Murashige and Skoog medium with Minimal Organics
OD_600_	Optical Density at 600 nm
PMSF	Phenyl Methyl Sulfonyl Fluoride
*p*NP-α-D-Gal*p*	*p*-4-nitrophenol-α-D-Galactopyranoside
POI	Protein of Interest
PROSS	Protein Repair One-Stop Shop
PSB-D	Plant Systems Biology—Dark
PTM	Post-Translational Modification
RSCU	Relative Synonymous Codon Usage
RFP	Red Fluorescent Protein
RMSD	Root-Mean Square Deviation
TCA	Trichloroacetic acid
TEV	Tobacco Etch Virus
TGH	Tris-Glycerol-HEPES
TRX	Thioredoxin
