# Supplementary material for: The Achilles Heel of Protein Biochemistry: Insolubility of Recombinant Proteins—A Case Study About Producing a Rice Enzyme
Source: Int J Mol Sci. 2025 Sep 15;26(18):8974. doi: 10.3390/ijms26188974 (PMC12470104; doi:10.3390/ijms26188974)

Supplementary File S3 – Analysis of codon bias for expression of OsAPSE in *E. coli*

Analysis of rare codons for expression in *E. coli*

https://people.mbi.ucla.edu/sumchan/caltor.html

Total number of codons: 649

Total number of nucleotides: 1947

ATG GGA AGG GGA GCC CCA TCC TCC CAT CCG CCG CCA TGG CGC CGC CTC CTC CGC TAC GCC CTC CTC TGC GCC CTC CTC CCC CCC TGG GGC ACT TCC GAG GCT AAT GAA CAA CTT GCT GAG TTT CCA CCA AGA GGC TGG AAT TCC TAT GAT TCC TTT TCA TGG AUA GTT GAT GAA AAT ACA TAC ATG CAA AAT GCG GAG ATC TTG GCA GAA AAA TTG CTC CCA CAT GGA TAT GAG TTT GCA GTT ATT GAT TAC CTC TGG TAC CGA AAG TAT GTT CAT GGG GCA TAC ACA GAT TCA TAT GGA TTT GAT AAC ATT GAT GAG TGG GGT CGG CCA TTT CCT GAT CTT CAA AGA TTT CCA TCA TCC AGA ATT GAT AAA GGG TTC AGT CAA CTT GCG AAC AAG GTG CAT GGA ATG GGC TTG AAA TTC GGA ATC CAT TTA ATG AAA GGG AUA AGT TTA CAG GCT GTG AAT GGA AAC ACA CCC AUA TTG GAC ATT AAA ACGGGA AAA CCC TAC GTA GAG GAT GGC CGG CAA TGG ACA GCT CGT GAT AUA GGT CTT ACA CAT AGA ACA TGT GCA TGG ATG CCA CAT GGA TTT ATG AGT GTA AAT ACT GAT ATT GGA GCT GGA AAG GCC TTC CUA AGA TCT CTT TAT CAA CAG TAC GCT GAT TGG GGT GTT GAT TTT GTG AAG GTT GAT TGT ATC TTC GGT ACG GAT TAC AGC CCA AAA GAA ATC AUA ACT ATT TCA GAG CTC TTG GCA GAG CTT GAC CGC CCC ATC ATC CTG TCC ATC TCA CCA GGA ACC GAA GTG ACT CCA GCA TTA GCC AAA AAC ATC AGT CAA CAT GTT AAC ATG TAC AGG AUA ACA GGG GAT GAT TGG GAC AAC TGG AAG GAT GTT AGT TCA CAT TTT GAC GTG TCT AGT TCC TTT GCT GCT GCA AAT AAA ATT GGG GCC AUA GGA TTA CGA GGA AGA TCT TGG CCA GAT TTA GAC ATG CTC CCA TTT GGC TGG CTT ACA AAT GCA GGT GTC AAT CAG GGT CCA CAT AGG CAA TGT GAA CTT ACA TCT GAT GAA CAG AGA ACA CAG AUA GCA CTT TGG TCA ATG GCT AAG TCT CCT CUA ATG TAT GGA GGA GAT TTG AGG CAT CTC GAC AAT GAC ACG TTA AGC AUA AUA ACA AAT CCT ACA TTA CTG AAA AUA AAT CAC TAC AGC AUA AAT AAT ATG GAG TTC CAT CAT GTG CAC AGT GAA AGG ACT TCC AAA GAA GAC AAG CAT TCT AGT CGT TTC AUA TCC GAA GAT CTT GTA CAC GTA CCA AAG ATT GAT GGT GTA TCT CTT GGT CTC ACT GCC TGC AGT GAT GAC AAA GCA AAT GGA TGG TAT ATG TTT TCA CAA CAT GGT AAA TCA GAT CAT AUA TGC AGG AAC TAT GGG ATG CAG GAT GAC AAA AAT ATC TCA TTT TGC CTG GGC AAA ACA ATT CCT CTC CTG ACA TCG GAT GAT AUA ATC GTA CAT AAT GAA GAA TAC CAA ACA AAG TTT CAC CTG GCA AAT ATG GAC AGT GAC GAT GCT TGT CTG GAT GCA TCT GGC AGT CAA CGG AGG ACA TCC TCA GAT AGC AAG TTT CCC ATG TTT TCA AGG TGC AGG TGG CAT GCT ATG CAG ATG TGG GAG CTG AAT GAG AAA GGA AAC CTC ATT AGC AGT TAC TCA AGA TTA TGT GCC ACG GTG GAA TCC AAC AAT AAA GGAGTT GTA ACT ACA GGA GCA GTA GCA CGT GCA TGG AUA GCA ACT GGG AGT AAA GGA GAA AUA TAC CTG GCG TTC TTC AAC CTT GAC TCC ATG AGC AGGAAG AUA ACC GCG AGA AUA TCA GAC CTG GAA AAG GTT CTC GGG AGT ACA TTC AUA AGA AAA GAC ACG TGC AGC TGC ACT GAA GTT TGG AGC GGG AGGAAT TTC GGT CGT GTG GAG GAA GAG ATT TCA GCG GTA GTT AAA TCA CAT GGT TCC ATG GTG TTT GAA ATT ACA TGT

Amino acid	Codon	Count	Frequency (%)
Arginine	CGA	2	0.3082
	CGG	3	0.4622
	AGG	11	1.6949
	AGA	10	1.5408
Glycine	GGA	21	3.2357
	GGG	9	1.3867
Isoleucine	AUA	20	3.0817
Leucine	CUA	2	0.3082
Proline	CCC	6	0.9245
Threonine	ACG	5	0.7704
**Total**		89	13.7134

Analysis of codon adaptation

https://www.biologicscorp.com/tools/RareCodonAnalyzer/

CAI index for expression of **native OsAPSE** in *E. coli*: **0.54**

CAI index for expression of **codon-optimized OsAPSE** in *E. coli*: **0.89**

Codon optimization (GeneArt)

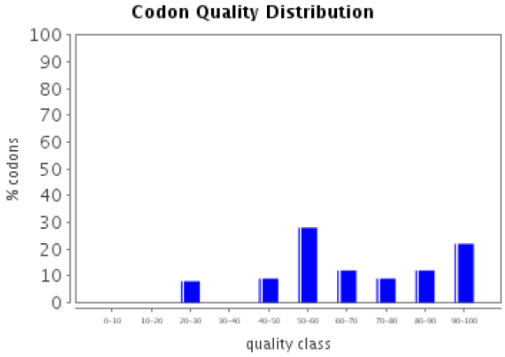

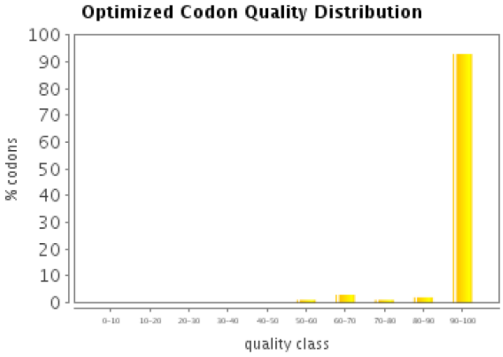

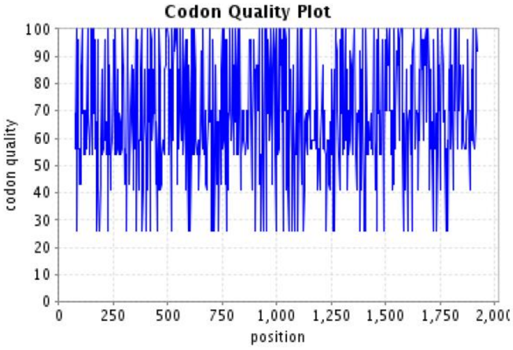

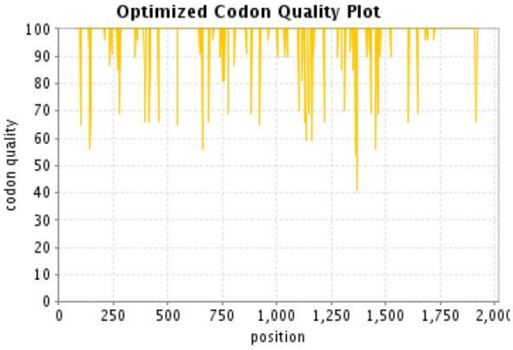

Supplement: Supplementary file 1 [file ijms-26-08974-s001.zip › ijms-3808161 -S3.pdf]
