# Supplementary material for: The Achilles Heel of Protein Biochemistry: Insolubility of Recombinant Proteins—A Case Study About Producing a Rice Enzyme
Source: Int J Mol Sci. 2025 Sep 15;26(18):8974. doi: 10.3390/ijms26188974 (PMC12470104; doi:10.3390/ijms26188974)
Supplement: Supplementary file 1 [file ijms-26-08974-s001.zip › ijms-3808161 -S6.pdf]

Supplementary File S6 – Comparison of AtAPSE and OsAPSE

Sequence identity

CLUSTAL O(1.2.4) multiple sequence alignment

```
AtAPSE      -MTGLSVEKTGSTRSTVFFIIFNLSIFSLSIEARSRQQHASFPPRGWNSYDSFCWTISEA 59
OsAPSE      MGRGAPSSHPPPWRRLLRYALL-CALLPPWGTSEANEQLAEFPPRGWNSYDSFSWIVDEN 59
               *   .:    *  : : ::  :::     :.:.:* *.************.* :.*

AtAPSE      EFLQSAEIVSKRLLPHGYQYVVVDYLWYRKKVEGAYVDSLGFDVIDEWGRLHPDPGRWPS 119
OsAPSE      TYMQNAEILAEKLLPHGYEFAVIDYLWYRKYVHGAYTDSYGFDNIDEWGRPFPDLQRFPS 119
             ::*.***::::******::.*:******* *.***.** *** ****** .**  *:**

AtAPSE      SRGGKGFTEVAEKVHRMGLKFGIHVMGGISTQAYNANSLVMDSVKGGAYEESGRQWRAKD 179
OsAPSE      SRIDKGFSQLANKVHGMGLKFGIHLMKGISLQAVNGNTPILDIKTGKPYVEDGRQWTARD 179
            ** .***:::*:*** ********:* *** ** *.*: ::*  .*  * *.**** *:*

AtAPSE      IGIKERACVWMSHGFMSVNTKLGAGKAFLRSLYRQYAEWGVDFIKHDCVFGTDFNIEEIT 239
OsAPSE      IGLTHRTCAWMPHGFMSVNTDIGAGKAFLRSLYQQYADWGVDFVKVDCIFGTDYSPKEII 239
            **:..*:*.** ********.:***********:***:*****:* **:****:. :**

AtAPSE      YVSEVLKELDRPVLYSISPGTSVTPTMAKEVSQLVNMYRITGDDWDTWKDVTAHFDISRD 299
OsAPSE      TISELLAELDRPIILSISPGTEVTPALAKNISQHVNMYRITGDDWDNWKDVSSHFDVSSS 299
             :**:* *****:: ******.***::**::** ************.****::***:* .

AtAPSE      LSASSMIGARGLQGKSWPDLDMLPLGWLTDQGSNVGPHRACNLNLEEQKSQMTLWSIAKS 359
OsAPSE      FAAANKIGAIGLRGRSWPDLDMLPFGWLTNAGVNQGPHRQCELTSDEQRTQIALWSMAKS 359
            ::*:. *** **:*:*********:****: * * **** *:*. :**::*::***:***

AtAPSE      PLMFGGDVRNLDATTYNLITNPTLLEINSYSSNNKEFPYITATRVSRNKHKGYPHH---P 416
OsAPSE      PLMYGGDLRHLDNDTLSIITNPTLLKINHYSINNMEFHHVHSERTSKEDKHSSRFISEDL 419
            ***:***:*:**  * .:*******:** ** ** ** :: : *.*::.::.  .

AtAPSE      TGKNISTKHAFGLTSCKEQKANTWFIVDKN--RGQICWNQHSSEKLEKPFCLYNRKALLA 474
OsAPSE      VHVPKIDGVSLGLTACSDDKANGWYMFSQHGKSDHICRNYGMQDDKNISFCLGKTIPLLT 479
            .        ::***:*.::*** *::..::   .:** *   .:. :  *** :   **:

AtAPSE      SDKKLKHNQLYQGKLHLHTNDKAQSCLAASSQQKLTSKDYSQGALSPCKLDANQMWELHS 534
OsAPSE      SDDIIVHNEEYQTKFHLANMDSDDACLDASGSQRRTSSDSKFPMFSRCRWHAMQMWELNE 539
            **. : **: ** *:** . *. ::** **..*: **.* .   :* *: .* *****:.

AtAPSE      NGTLENSYSGLCAVLNPVKAAE-ASSNGVRSWIATGRRGEVYVAFFNLNQEKTKISAKIS 593
OsAPSE      KGNLISSYSRLCATVESNNKGVVTTGAVARAWIATGSKGEIYLAFFNLDSMSRKITARIS 599
            :*.* .*** ***.::  : .  ::.  .*:***** :**:*:*****:. . **:*:**

AtAPSE      DIATALRGKKNLVGASCTSHELWSGKDFGPTKDSVSIQVEPHGPALFVLHCSNA 647
OsAPSE      DLEKVL-GSTFIRKDTCSCTEVWSGRNFGRVEEEISAVVKSHGSMVFEITC--- 649
            *: ..* *.. :   :*:. *:***::** .::.:*  *: **  :* : *
```

Symbols: identical residue (*), highly conserved residue (:), conserved residue (.), gap (–).

Structural alignment (PyMol) of AlphaFold models

The AlphaFold structures of **AtAPSE** (UniProt ID: F4JCI4) and **OsAPSE** (UniProt: Q5QLK3) were obtained via UniProt.

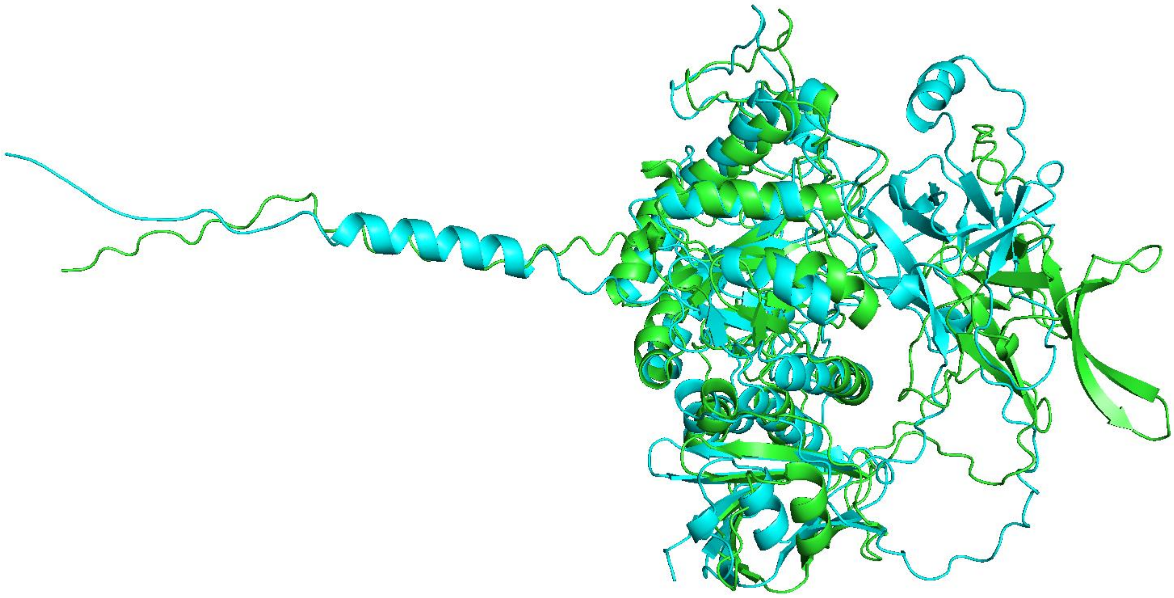

Discussion of differences and resemblances between AtAPSE and OsAPSE

Redrafted from PhD dissertation (p. 205-207), with permission of all authors [1].

OsAPSE was named after its *Arabidopsis* homolog AtAPSE. Indeed, AtAPSE and OsAPSE are phylogenetically related and are present in the same 'plant β-L-arabinopyranosidase subfamily', as reported by Imaizumi *et al*., (2017), which suggests that AtAPSE and OsAPSE might share biochemical properties and biological function. There are, however, several important differences between AtAPSE and OsAPSE [2].

AtAPSE was produced recombinantly in *Pichia pastoris* and it was demonstrated that this enzyme only possesses ARAP activity on synthetic and natural substrates. This is in contrast to OsAPSE, which displays both AGAL and ARAP activity. Imaizumi *et al*., (2017) attribute the fact that AtAPSE only displays ARAP activity to the presence of a tyrosine residue in the catalytic pocket of AtAPSE, whereas regular AtAGALs have an aspartic residue (not the catalytic residue). They hypothesize that the presence of a bulkier tyrosine residue allows only binding to L-Ara*p* and does not allow binding to D-Gal*p*, while the aspartic acid residue in AtAGALs is less bulky and allows substrate binding to the C6 hydroxyl group of D-Gal*p* instead. However, our molecular docking and dynamics analyses have shown that the relevant tyrosine residues in OsAPSE are not directly involved in substrate binding, but assist in binding through stacking interactions (more distant interactions) rather than direct hydrogen bonding with the substrate [3]. In their discussion section, Imaizumi *et al*., (2017) admit that the carbohydrate recognition specificity of GH27 enzymes is not solely attributable to the presence of either a tyrosine or an aspartic acid residue, and that also other residues affect the substrate specificity towards D-Gal*p* and L-Ara*p*.

Imaizumi *et al*., (2017) also investigated the properties of a series of other AGALs from *Arabidopsis*. AtAGAL1 was not investigated by Imaizumi *et al*., (2017) as this was already done by Tapernoux-Lüthi *et al*., (2004). It was demonstrated that AtAGAL2 and AtAGAL3 display both dual ARAP and AGAL activity, albeit to different extents. This could suggest that also other AGALs in

rice would display multiple activities and could replace OsAPSE in *osapse* plants. The phylogenetic similarities between AGALs and APSEs from *Arabidopsis* and rice were studied and reported in **Figure S5.1**, and showed that paralogous GH27 AtAGALs and AtAPSE are orthologous towards OsAGALs and OsAPSE from rice (**Table S5.1**).

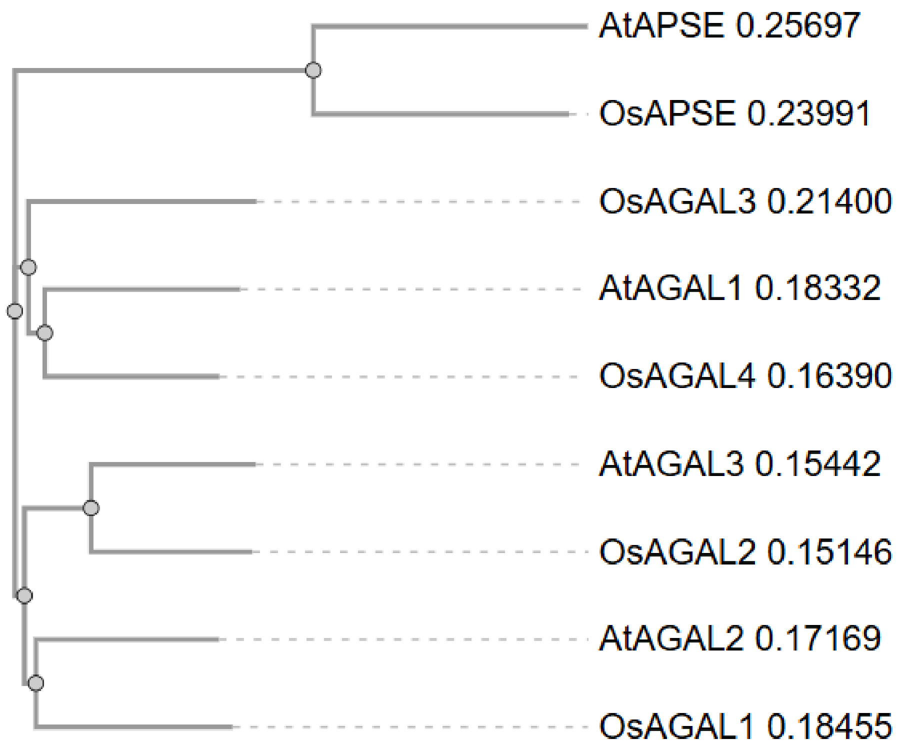

Figure S5.1 – Phylogenetic tree displaying the sequence similarities between AGALs and ARAPs from *Arabidopsis* and rice. The phylogenetic tree was constructed using the Neighbor-Joining algorithm in Clustal Omega. The branch lengths display distance scores next to the *Arabidopsis* or rice enzymes.

Since phylogenetic similarity suggests structural similarity, and structural similarity implies functional similarity [4], it was investigated to what extent the activities between the *Arabidopsis* and rice homologues would be similar, based on literature findings. **It was found that homologous enzymes did not necessarily display identical activities** (**Table S5.1**). For instance: AtAGAL2 displays mainly AGAL activity, but also ARAP and ARAF (α-L-arabinofuranosidase) to a lesser extent, while its closest homolog in rice (OsAGAL1) is restricted to AGAL activity. Moreover, AtAGAL3 displays both AGAL and ARAP activity, although its rice homolog (OsAGAL2) is only reported to display AGAL activity. The homolog of AtAGAL1 in rice (OsAGAL4) has not been characterized yet, and for OsAGAL3 there is no homolog in *Arabidopsis*. In contrast to AGALs from *Arabidopsis*, OsAGALs do not display ARAP activity – or it has never been investigated before.

Table S5.1 – Comparison of enzymatic and biochemical properties of AGAL and APSE proteins from *Arabidopsis* and rice

***Arabidopsis thaliana* (thale cress)**	Enzyme	**AtAGAL1**	**AtAGAL2**	**AtAGAL3**	**AtAPSE**	
	Locus	At5g08380.1	At5g08370.1	At3g56310.1	At3g26380.1	
	Theoretical size (kDa)	45.7	44.0	48.4	72.3	
	Activities	AGAL	AGAL (100%), ARAP (10.6%), ARAF (0.2%)	AGAL (100%), ARAP (7%)	ARAP (100%)	
	Natural substrates	RFOs	Arabinogalactan	Galactomannan, galactoglucomannan	Arabinogalactan, gum arabic	
	Source	[8]	[2]	[2]	[2]	
***Oryza sativa* (rice)**	Homologous enzyme	**OsAGAL4**	**OsAGAL1**	**OsAGAL2**	**OsAPSE**	**OsAGAL3**
	Locus	LOC_Os10g35070	LOC_Os10g35110	LOC_Os07g48160	LOC_Os01g33420	LOC_Os07g26900
	Sequence identity to closest homolog	65.3	64.4	69.4	50.3	*n.a.*
	Theoretical size (kDa)	31.4	45.8	47.0	73.2	43.7
	Activities	*n.a.*	AGAL	AGAL	ARAP (100%), AGAL (85.9%)	AGAL
	Natural substrates	*n.a.*	RFOs, guar gum, galactomannan	RFOs	Arabinogalactan, RFOs, AGP *O*-glycans, galacto-mannan, melibiose	RFOs
	Source	*n.a.*	[6,7]	[5]	[3]	[5]

Abbreviations: *n.a.* (not analyzed)

<u>References</u>

1. De Coninck, T. Biochemical and Biological Properties of a Bifunctional GH27 Enzyme from Rice. PhD dissertation, Universiteit Gent (Ghent University): Gent, Belgium, 2025.
2. Imaizumi, C.; Tomatsu, H.; Kitazawa, K.; Yoshimi, Y.; Shibano, S.; Kikuchi, K.; Yamaguchi, M.; Kaneko, S.; Tsumuraya, Y.; Kotake, T. Heterologous Expression and Characterization of an Arabidopsis β-L-Arabinopyranosidase and α-D-Galactosidases Acting on β-L-Arabinopyranosyl Residues. *Journal of Experimental Botany* **2017**, *68*, 4651–4661, doi:10.1093/jxb/erx279.
3. De Coninck, T.; Verbeke, I.; Rougé, P.; Desmet, T.; Van Damme, E.J.M. OsAPSE Modulates Non-Covalent Interactions between Arabinogalactan Protein O-Glycans and Pectin in Rice Cell Walls. *Frontiers in Plant Science* **2025**, *16*, doi:10.3389/fpls.2025.1588802.
4. Illergård, K.; Ardell, D.H.; Elofsson, A. Structure Is Three to Ten Times More Conserved than Sequence—A Study of Structural Response in Protein Cores. *Proteins* **2009**, *77*, 499–508, doi:10.1002/prot.22458.
5. Li, S.; Kim, W.-D.; Kaneko, S.; Prema, P.A.; Nakajima, M.; Kobayashi, H. Expression of Rice (*Oryza Sativa* L. Var. Nipponbare) α-Galactosidase Genes in *Escherichia Coli* and Characterization. *Bioscience, Biotechnology, and Biochemistry* **2007**, *71*, 520–526, doi:10.1271/bbb.60554.
6. Kim, W.-D.; Kobayashi, O.; Kaneko, S.; Sakakibara, Y.; Park, G.-G.; Kusakabe, I.; Tanaka, H.; Kobayashi, H. A-Galactosidase from Cultured Rice (Oryza Sativa L. Var. Nipponbare) Cells. **2002**.
7. Fujimoto, Z.; Kaneko, S.; Momma, M.; Kobayashi, H.; Mizuno, H. Crystal Structure of Rice α-Galactosidase Complexed with D-Galactose. *Journal of Biological Chemistry* **2003**, *278*, 20313–20318, doi:10.1074/jbc.M302292200.
8. Tapernoux-Lüthi, E.M.; Böhm, A.; Keller, F. Cloning, Functional Expression, and Characterization of the Raffinose Oligosaccharide Chain Elongation Enzyme, Galactan:Galactan Galactosyltransferase, from Common Bugle Leaves. *Plant Physiology* **2004**, *134*, 1377–1387, doi:10.1104/pp.103.036210.
