# Supplementary material for: The Achilles Heel of Protein Biochemistry: Insolubility of Recombinant Proteins—A Case Study About Producing a Rice Enzyme
Source: Int J Mol Sci. 2025 Sep 15;26(18):8974. doi: 10.3390/ijms26188974 (PMC12470104; doi:10.3390/ijms26188974)
Supplement: Supplementary file 1 [file ijms-26-08974-s001.zip › ijms-3808161 -S7.pdf]

## Supplementary File S7 – OsAPSE sequence and entries in databases

| Database | entry |
|---|---|
| Locus ID | LOC_Os01g33420 |
| GenBank ID | BAD73696.1 |
| UniProt ID | Q5QLK3 |

**>LOC_Os01g33420.1**

ATGGGAAGGGGAGCCCCATCCTCCCATCCGCCGCCATGGCGCCGCCTCCTCCGCTACGCC
CTCCTCTGCGCCCTCCTCCCCCCCTGGGGCACTTCCGAGGCTAATGAACAACTTGCTGAG
TTTCCACCAAGAGGCTGGAATTCCTATGATTCCTTTTCATGGATAGTTGATGAAAATACA
TACATGCAAAATGCGGAGATCTTGGCAGAAAAATTGCTCCCACATGGATATGAGTTTGCA
GTTATTGATTACCTCTGGTACCGAAAGTATGTTCATGGGGCATACACAGATTCATATGGA
TTTGATAACATTGATGAGTGGGGTCGGCCATTTCCTGATCTTCAAAGATTTCCATCATCC
AGAATTGATAAAGGGTTCAGTCAACTTGCGAACAAGGTGCATGGAATGGGCTTGAAATTC
GGAATCCATTTAATGAAAGGGATAAGTTTACAGGCTGTGAATGGAAACACACCCATATTG
GACATTAAAACGGGAAAACCCTACGTAGAGGATGGCCGGCAATGGACAGCTCGTGATATA
GGTCTTACACATAGAACATGTGCATGGATGCCACATGGATTTATGAGTGTAAATACTGAT
ATTGGAGCTGGAAAGGCCTTCCTAAGATCTCTTTATCAACAGTACGCTGATTGGGGTGTT
GATTTTGTGAAGGTTGATTGTATCTTCGGTACGGATTACAGCCCAAAAGAAATCATAACT
ATTTCAGAGCTCTTGGCAGAGCTTGACCGCCCCATCATCCTGTCCATCTCACCAGGAACC
GAAGTGACTCCAGCATTAGCCAAAAACATCAGTCAACATGTTAACATGTACAGGATAACA
GGGGATGATTGGGACAACTGGAAGGATGTTAGTTCACATTTTGACGTGTCTAGTTCCTTT
GCTGCTGCAAATAAAATTGGGGCCATAGGATTACGAGGAAGATCTTGGCCAGATTTAGAC
ATGCTCCCATTTGGCTGGCTTACAAATGCAGGTGTCAATCAGGGTCCACATAGGCAATGT
GAACTTACATCTGATGAACAGAGAACACAGATAGCACTTTGGTCAATGGCTAAGTCTCCT
CTAATGTATGGAGGAGATTTGAGGCATCTCGACAATGACACGTTAAGCATAATAACAAAT
CCTACATTACTGAAAATAAATCACTACAGCATAAATAATATGGAGTTCCATCATGTGCAC
AGTGAAAGGACTTCCAAAGAAGACAAGCATTCTAGTCGTTTCATATCCGAAGATCTTGTA
CACGTACCAAAGATTGATGGTGTATCTCTTGGTCTCACTGCCTGCAGTGATGACAAAGCA
AATGGATGGTATATGTTTTCACAACATGGTAAATCAGATCATATATGCAGGAACTATGGG
ATGCAGGATGACAAAAATATCTCATTTTGCCTGGGCAAAACAATTCCTCTCCTGACATCG
GATGATATAATCGTACATAATGAAGAATACCAAACAAAGTTTCACCTGGCAAATATGGAC
AGTGACGATGCTTGTCTGGATGCATCTGGCAGTCAACGGAGGACATCCTCAGATAGCAAG
TTTCCCATGTTTTCAAGGTGCAGGTGGCATGCTATGCAGATGTGGGAGCTGAATGAGAAA
GGAAACCTCATTAGCAGTTACTCAAGATTATGTGCCACGGTGGAATCCAACAATAAAGGA
GTTGTAACTACAGGAGCAGTAGCACGTGCATGGATAGCAACTGGGAGTAAAGGAGAAATA
TACCTGGCGTTCTTCAACCTTGACTCCATGAGCAGGAAGATAACCGCGAGAATATCAGAC
CTGGAAAAGGTTCTCGGGAGTACATTCATAAGAAAAGACACGTGCAGCTGCACTGAAGTT
TGGAGCGGGAGGAATTTCGGTCGTGTGGAGGAAGAGATTTCAGCGGTAGTTAAATCACAT
GGTTCCATGGTGTTTGAAATTACATGTTGA

**>OsAPSE**

MGRGAPSSHPPPWRRLLRYALLCALLPPWGTSEANEQLAEFPPRGWNSYDSFSWIVDENT
YMQNAEILAEKLLPHGYEFAVIDYLWYRKYVHGAYTDSYGFDNIDEWGRPFPDLQRFPSS
RIDKGFSQLANKVHGMGLKFGIHLMKGISLQAVNGNTPILDIKTGKPYVEDGRQWTARDI
GLTHRTCAWMPHGFMSVNTDIGAGKAFLRSLYQQYADWGVDFVKVDCIFGTDYSPKEIIT
ISELLAELDRPIILSISPGTEVTPALAKNISQHVNMYRITGDDWDNWKDVSSHFDVSSSF
AAANKIGAIGLRGRSWPDLDMLPFGWLTNAGVNQGPHRQCELTSDEQRTQIALWSMAKSP
LMYGGDLRHLDNDTLSIITNPTLLKINHYSINNMEFHHVHSERTSKEDKHSSRFISEDLV
HVPKIDGVSLGLTACSDDKANGWYMFSQHGKSDHICRNYGMQDDKNISFCLGKTIPLLTS
DDIIVHNEEYQTKFHLANMDSDDACLDASGSQRRTSSDSKFPMFSRCRWHAMQMWELNEK
GNLISSYSRLCATVESNNKGVVTTGAVARAWIATGSKGEIYLAFFNLDSMSRKITARISD
LEKVLGSTFIRKDTCSCTEVWSGRNFGRVEEEISAVVKSHGSMVFEITC
