# Supplementary material for: The Achilles Heel of Protein Biochemistry: Insolubility of Recombinant Proteins—A Case Study About Producing a Rice Enzyme
Source: Int J Mol Sci. 2025 Sep 15;26(18):8974. doi: 10.3390/ijms26188974 (PMC12470104; doi:10.3390/ijms26188974)
Supplement: Supplementary file 1 [file ijms-26-08974-s001.zip › ijms-3808161 -S8.pdf]

Supplementary File S8 – Overview of the used oligonucleotides in this study.

Name	Orientation	Sequence	Purpose
Evd275	Fw	GCCTGAACACCATATCCATCC	Amplification of the pJET1.2 backbone
Evd276	Rv	GCAGCTGAGAATATTGTAGGAGATC	
Evd825	Fw	TAATACGACTCACTATAGGG	Amplification of the T7 promotor and terminator
Evd826	Rv	TAGTTATTGCTCAGCGGTGG	
Evd021	Fw	GACTGGTTCCAATTGACAAGC	Amplification of the AOX promotor and terminator
Evd022	Rv	GCAAATGGCATTCTGACATCC	
Evd386	Fw	GTAAAACGACGGCCAG	Amplification of the pK7WG2D backbone
Evd387	Rv	CAGGAAACAGCTATGAC	
L366	Fw	AAAAAGCAGGCTTCACCATGGGAAGGGGAGCCCCA	Addition of first ½ attB sites
L367	Rv	AGAAAGCTGGGTGACATGTAATTTCAAAC	
Evd002	Fw	GGGGACAAGTTTGTACAAAAAAGCAGGCT	Completion of attB sites
Evd004	Rv	GGGGACCACTTGTACAAGAAAGCTGGGT	
