# Supplementary material for: The Achilles Heel of Protein Biochemistry: Insolubility of Recombinant Proteins—A Case Study About Producing a Rice Enzyme
Source: Int J Mol Sci. 2025 Sep 15;26(18):8974. doi: 10.3390/ijms26188974 (PMC12470104; doi:10.3390/ijms26188974)
Supplement: Supplementary file 1 [file ijms-26-08974-s001.zip › ijms-3808161-S1.pdf]

Supplementary File S1 – Key characteristics of different hosts for recombinant protein production [1–4].

Criterion	Bacterial cells	Yeast/fungal cells	Plant cells	Insect cells	Mammalian cells
Common example	*Escherichia coli*	*Pichia pastoris*	*Arabidopsis* PSB-D	*Spodoptera frugiperda*	Chinese Hamster Ovaries
Ease of genetic manipulation	Easy	Moderate	Difficult	Moderate	Difficult
Time for protein production	1-2 days	3-5 days	weeks	7-10 days	Weeks to months
Cell doubling time	Very fast (20 minutes)	Moderate (1-2 hours)	Slow (20-48 hours)	Slow (18-24 hours)	Slow (16-24 hours)
Cultivation costs	Very low	Low to moderate	Moderate to high	High	Very high
Complexity of growth media	Simple	Moderate	Complex	Very complex	Very complex
Expression (mg protein per L of medium)	100-5000	100-5000	10-100	10-1000	10-1000
Scalability	High	High	Moderate	Moderate	High
Disulfide bridges	No	Yes	Yes	Yes	Yes
Glycosylation type	None	High-mannose *N*-glycans	Complex (plant-specific) *N*-glycans	Partial (insect-specific) *N*-glycans	Fully human-like *N*-glycans
Protein secretion	Periplasmic is possible but requires secretion signals	Possible	Possible	Possible	Possible
Protein stability and degradation risk	High – risk of inclusion bodies	Moderate – secreted proteins are more stable	Risk of proteolysis	Moderate	High – minimal proteolysis
Suitability for complex eukaryotic proteins	Usually not	Yes	Yes	Yes	Yes
Regulatory approval and industrial use	Widely used for research, not for therapeutics , except small proteins that do not require PTMs (f.i. insulin)	Approved for some enzymes and vaccines	Limited biopharma use	Used in some vaccines	Industry standards, FDA/EMA approved

References

1. Balen, B.; Krsnik-Rasol, M. N-Glycosylation of Recombinant Therapeutic Glycoproteins in Plant Systems. **2007**.
2. Karbalaei, M.; Rezaee, S.A.; Farsiani, H. *Pichia Pastoris* : A Highly Successful Expression System for Optimal Synthesis of Heterologous Proteins. *Journal Cellular Physiology* **2020**, *235*, 5867–5881, doi:10.1002/jcp.29583.
3. Zhang, T.; Liu, H.; Lv, B.; Li, C. Regulating Strategies for Producing Carbohydrate Active Enzymes by Filamentous Fungal Cell Factories. *Front. Bioeng. Biotechnol.* **2020**, *8*, 691, doi:10.3389/fbioe.2020.00691.
4. Schütz, A.; Bernhard, F.; Berrow, N.; Buyel, J.F.; Ferreira-da-Silva, F.; Haustraete, J.; van den Heuvel, J.; Hoffmann, J.-E.; de Marco, A.; Peleg, Y.; et al. A Concise Guide to Choosing Suitable Gene Expression Systems for Recombinant Protein Production. *STAR Protocols* **2023**, *4*, doi:10.1016/j.xpro.2023.102572.
