# Supplementary material for: The Achilles Heel of Protein Biochemistry: Insolubility of Recombinant Proteins—A Case Study About Producing a Rice Enzyme
Source: Int J Mol Sci. 2025 Sep 15;26(18):8974. doi: 10.3390/ijms26188974 (PMC12470104; doi:10.3390/ijms26188974)
Supplement: Supplementary file 1 [file ijms-26-08974-s001.zip › ijms-3808161-S10.pptx]

## Slide 1
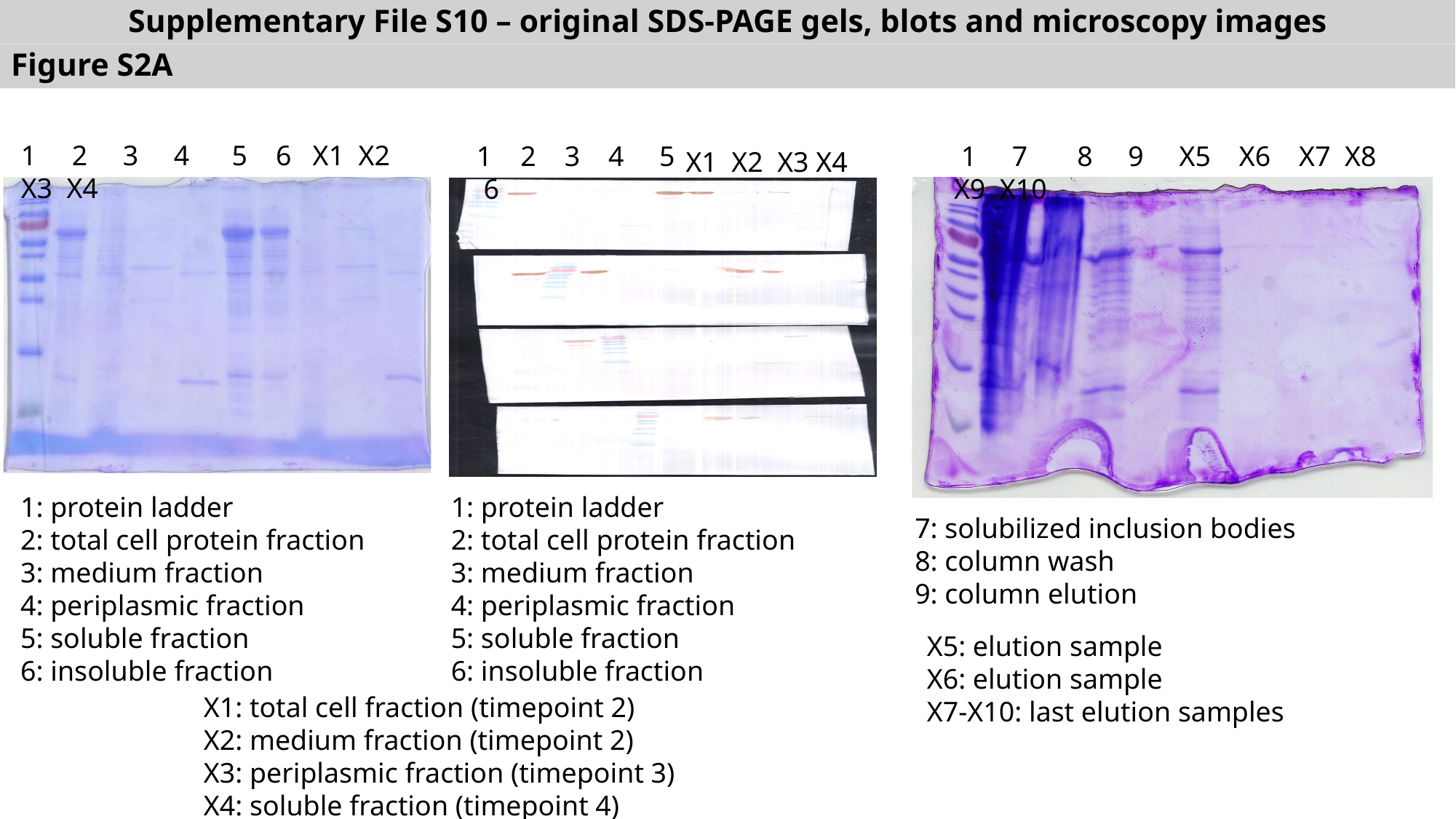

Supplementary File S10 – original SDS-PAGE gels, blots and microscopy images
Figure S2A
1 2 3 4 5 6 X1 X2 X3 X4
1 2 3 4 5 6
 1 7 8 9 X5 X6 X7 X8 X9 X10
X1 X2 X3 X4
1: protein ladder
2: total cell protein fraction
3: medium fraction
4: periplasmic fraction
5: soluble fraction
6: insoluble fraction
1: protein ladder
2: total cell protein fraction
3: medium fraction
4: periplasmic fraction
5: soluble fraction
6: insoluble fraction
7: solubilized inclusion bodies
8: column wash
9: column elution
X5: elution sample
X6: elution sample
X7-X10: last elution samples
X1: total cell fraction (timepoint 2)
X2: medium fraction (timepoint 2)
X3: periplasmic fraction (timepoint 3)
X4: soluble fraction (timepoint 4)

## Slide 2
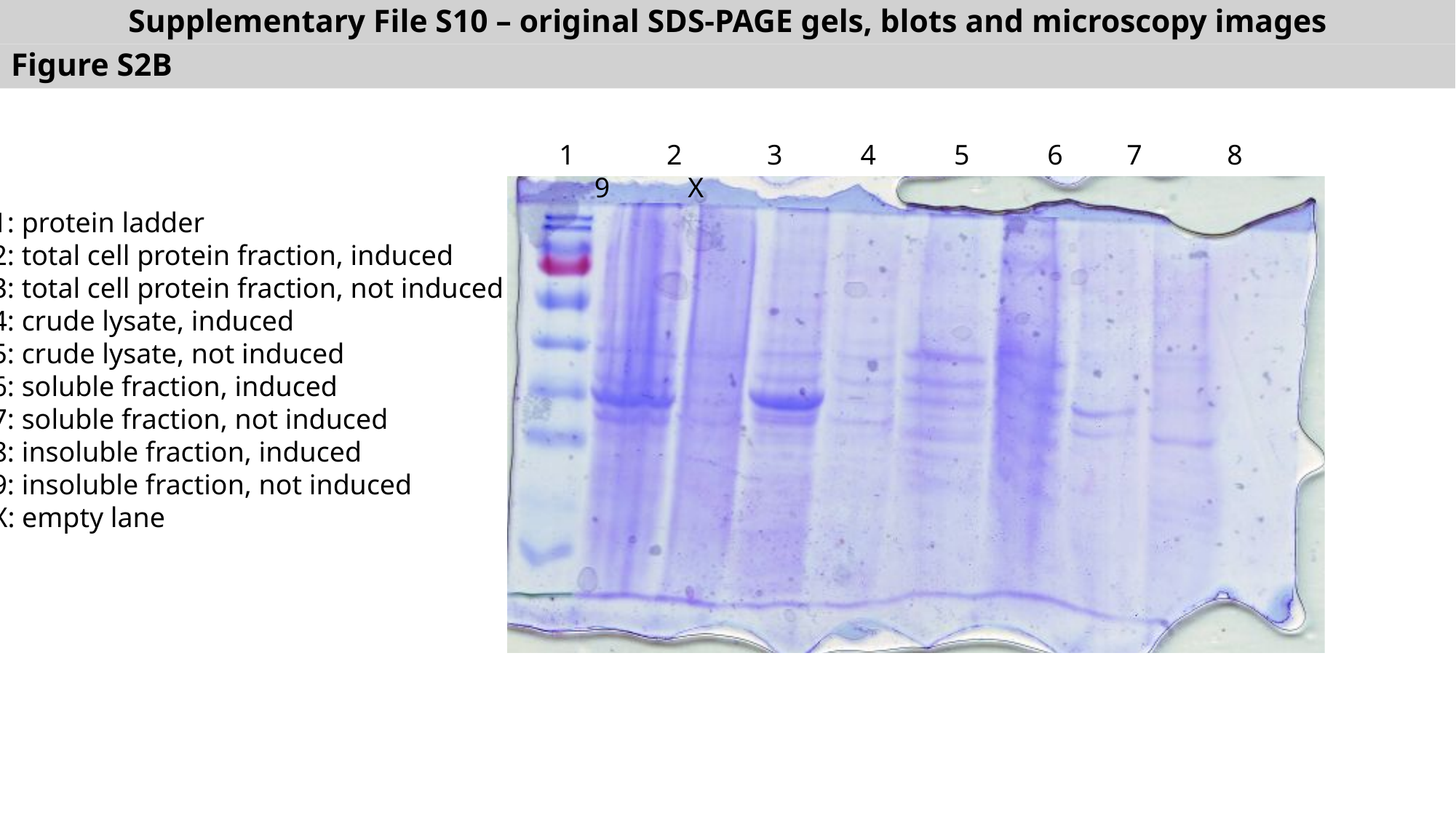

Supplementary File S10 – original SDS-PAGE gels, blots and microscopy images
Figure S2B
1 2 3 4 5 6 7 8 9 X
1: protein ladder
2: total cell protein fraction, induced
3: total cell protein fraction, not induced
4: crude lysate, induced
5: crude lysate, not induced
6: soluble fraction, induced
7: soluble fraction, not induced
8: insoluble fraction, induced
9: insoluble fraction, not induced
X: empty lane

## Slide 3
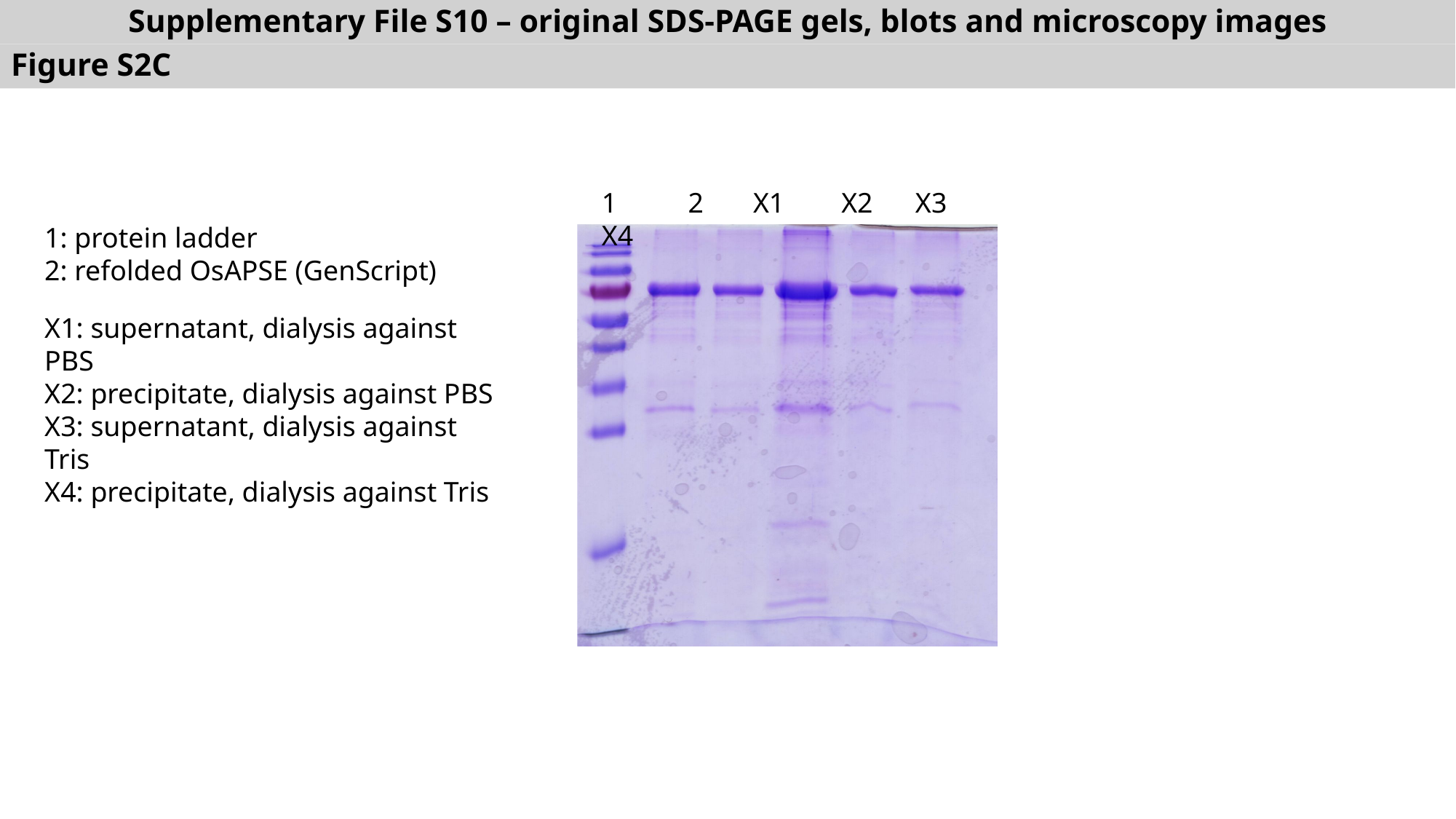

Supplementary File S10 – original SDS-PAGE gels, blots and microscopy images
Figure S2C
1 2 X1 X2 X3 X4
1: protein ladder
2: refolded OsAPSE (GenScript)
X1: supernatant, dialysis against PBS
X2: precipitate, dialysis against PBS
X3: supernatant, dialysis against Tris
X4: precipitate, dialysis against Tris

## Slide 4
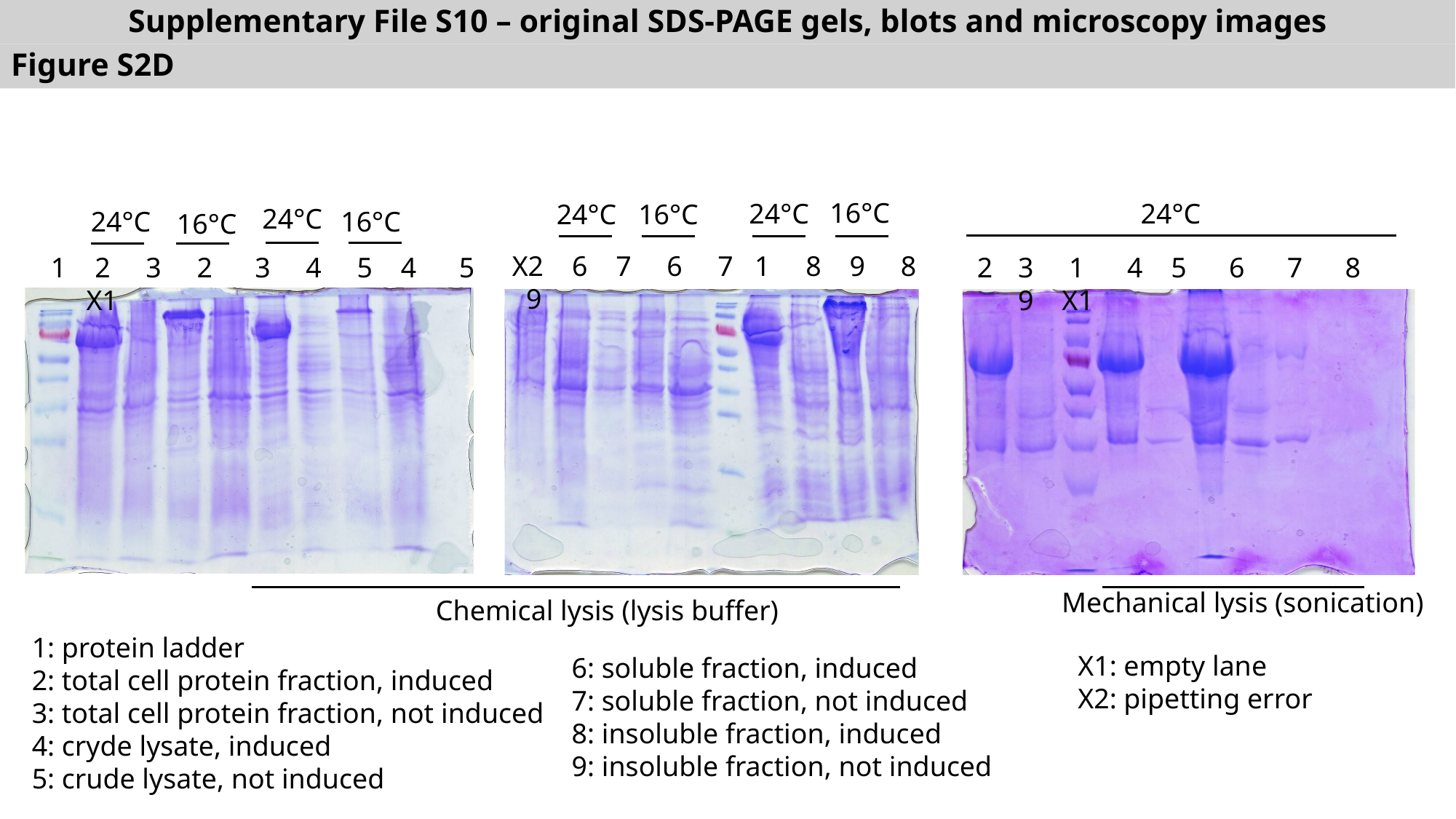

Supplementary File S10 – original SDS-PAGE gels, blots and microscopy images
Figure S2D
16°C
24°C
24°C
24°C
16°C
24°C
16°C
24°C
16°C
X2 6 7 6 7 1 8 9 8 9
1 2 3 2 3 4 5 4 5 X1
3 1 4 5 6 7 8 9 X1
Mechanical lysis (sonication)
Chemical lysis (lysis buffer)
1: protein ladder
2: total cell protein fraction, induced
3: total cell protein fraction, not induced
4: cryde lysate, induced
5: crude lysate, not induced
X1: empty lane
X2: pipetting error
6: soluble fraction, induced
7: soluble fraction, not induced
8: insoluble fraction, induced
9: insoluble fraction, not induced

## Slide 5
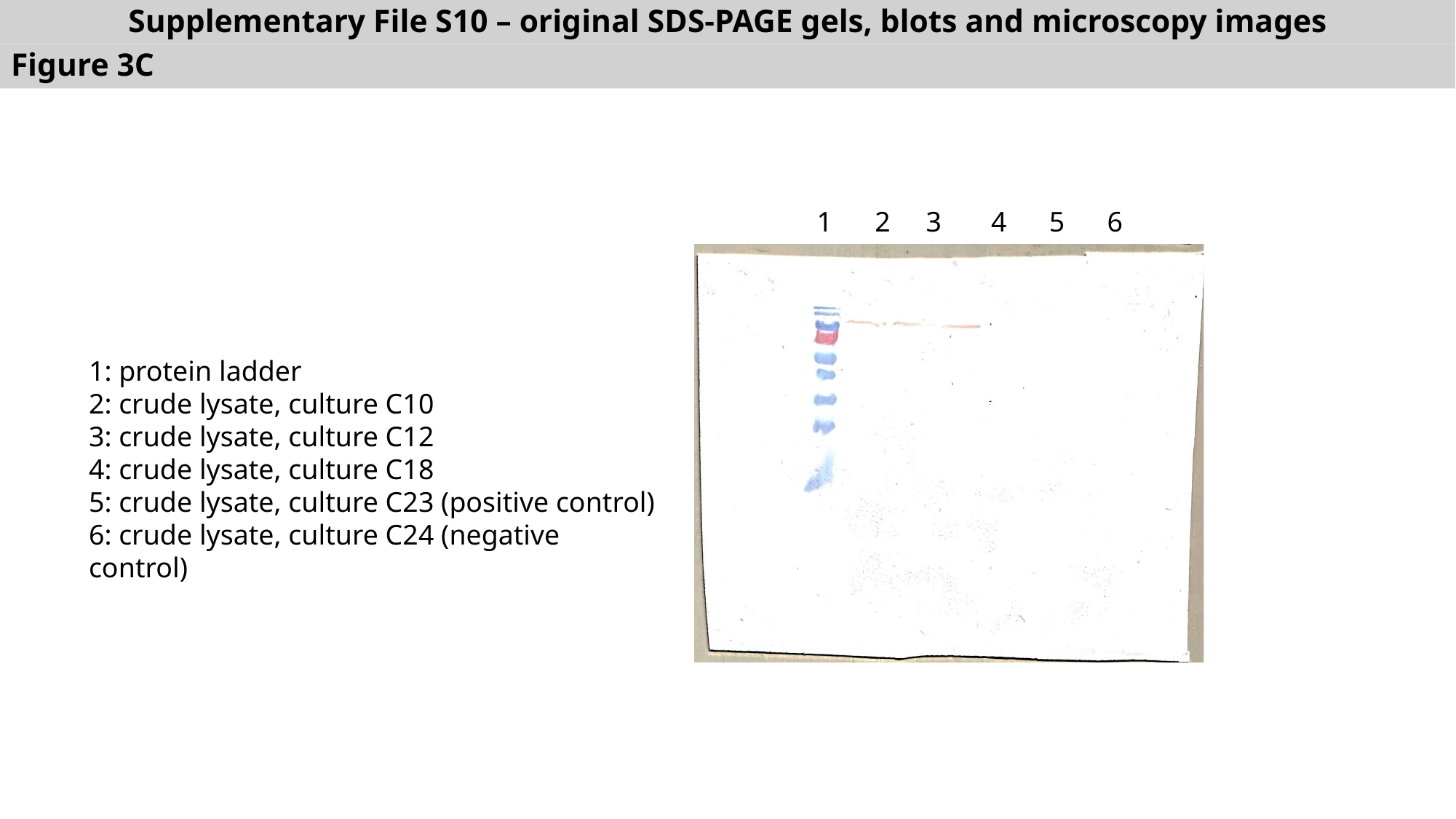

Supplementary File S10 – original SDS-PAGE gels, blots and microscopy images
Figure 3C
1 2 3 4 5 6
1: protein ladder
2: crude lysate, culture C10
3: crude lysate, culture C12
4: crude lysate, culture C18
5: crude lysate, culture C23 (positive control)
6: crude lysate, culture C24 (negative control)

## Slide 6
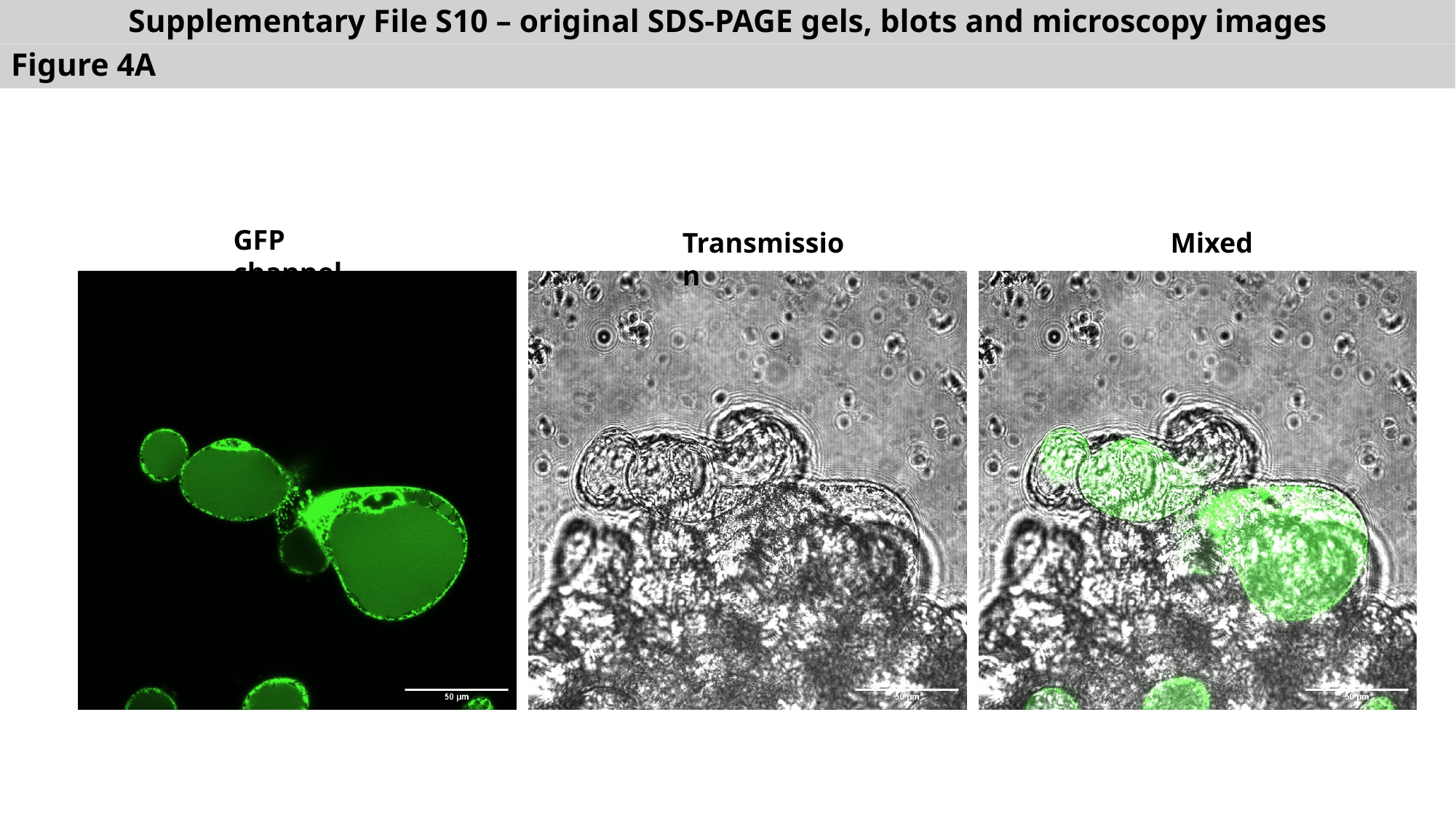

Supplementary File S10 – original SDS-PAGE gels, blots and microscopy images
Figure 4A
GFP channel
Transmission
Mixed

## Slide 7
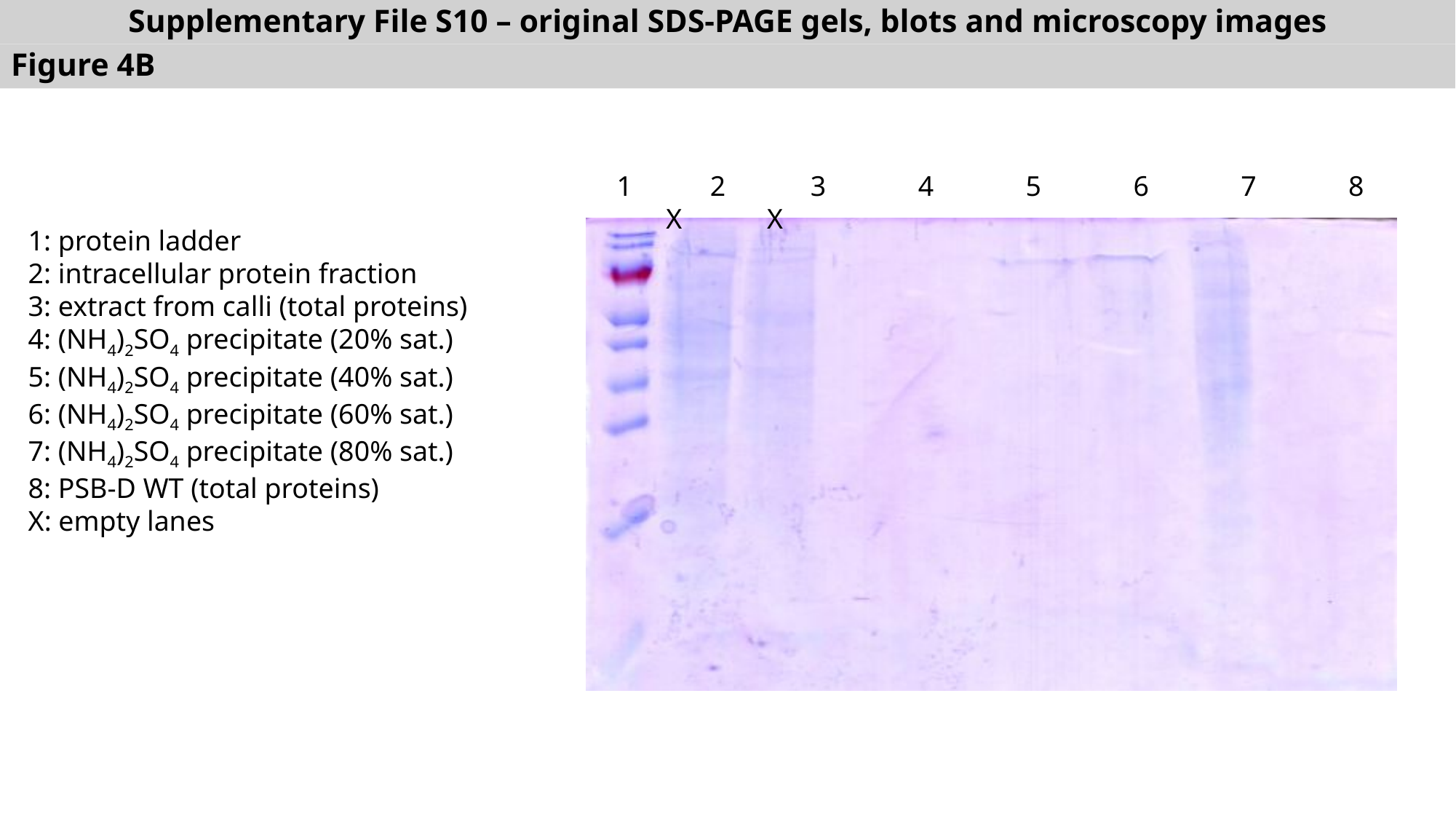

Supplementary File S10 – original SDS-PAGE gels, blots and microscopy images
Figure 4B
1 2 3 4 5 6 7 8 X X
1: protein ladder
2: intracellular protein fraction
3: extract from calli (total proteins)
4: (NH4)2SO4 precipitate (20% sat.)
5: (NH4)2SO4 precipitate (40% sat.)
6: (NH4)2SO4 precipitate (60% sat.)
7: (NH4)2SO4 precipitate (80% sat.)
8: PSB-D WT (total proteins)
X: empty lanes
