# Supplementary material for: The Achilles Heel of Protein Biochemistry: Insolubility of Recombinant Proteins—A Case Study About Producing a Rice Enzyme
Source: Int J Mol Sci. 2025 Sep 15;26(18):8974. doi: 10.3390/ijms26188974 (PMC12470104; doi:10.3390/ijms26188974)
Supplement: Supplementary file 1 [file ijms-26-08974-s001.zip › ijms-3808161-S2.pdf]

**Supplementary File S2 – Expression experiments in different *E. coli* strains usually resulted in insoluble protein of interest.**

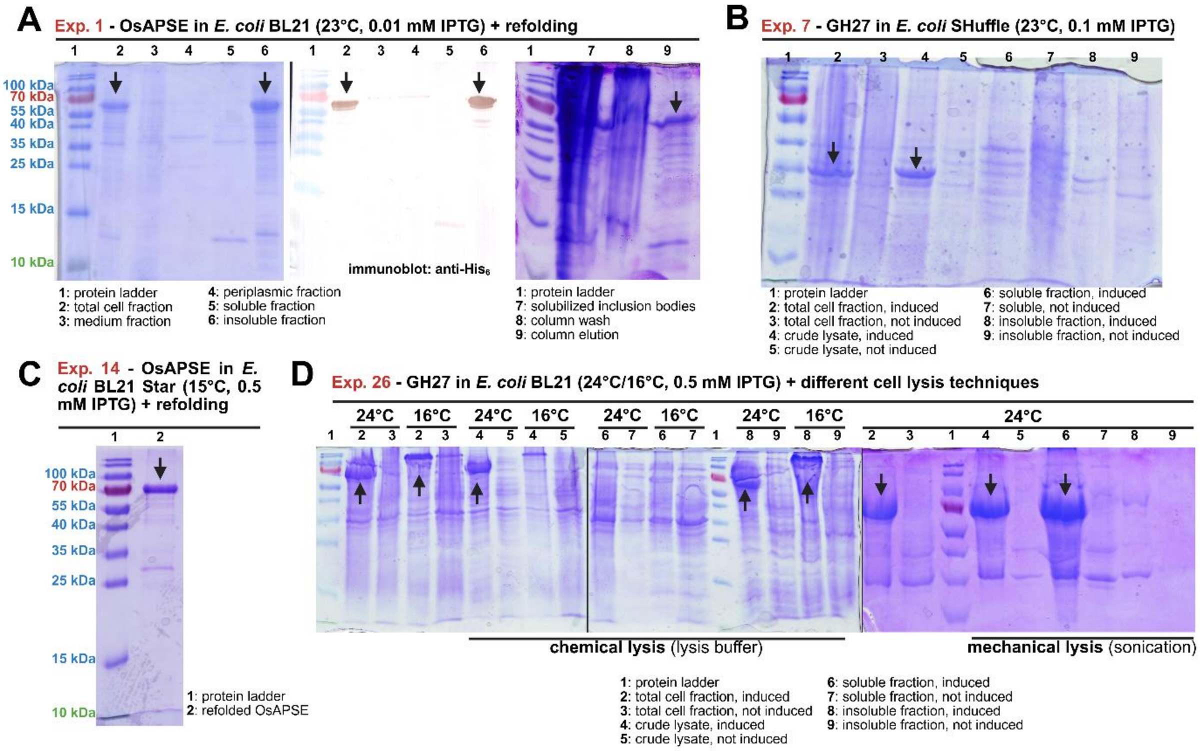


Results from experiment 1 (**A**), experiment 7 (**B**), experiment 14 (**C**) and experiment 26 (**D**) are shown. For experiment 26, protein samples were run across multiple gels. The contents of every lane is highlighted below or next to the SDS-PAGE gel or immunoblot. The protein of interest is indicated with an arrow.
